# Supplementary material for: Salinity tolerance loci revealed in rice using high-throughput non-invasive phenotyping
Source: Nat Commun. 2016 Nov 17;7:13342. doi: 10.1038/ncomms13342 (PMC5118543; doi:10.1038/ncomms13342)
Supplement: Supplementary Information — Supplementary Figure 1-10, Supplementary Tables 1-6 and Supplementary References. [file ncomms13342-s1.pdf]

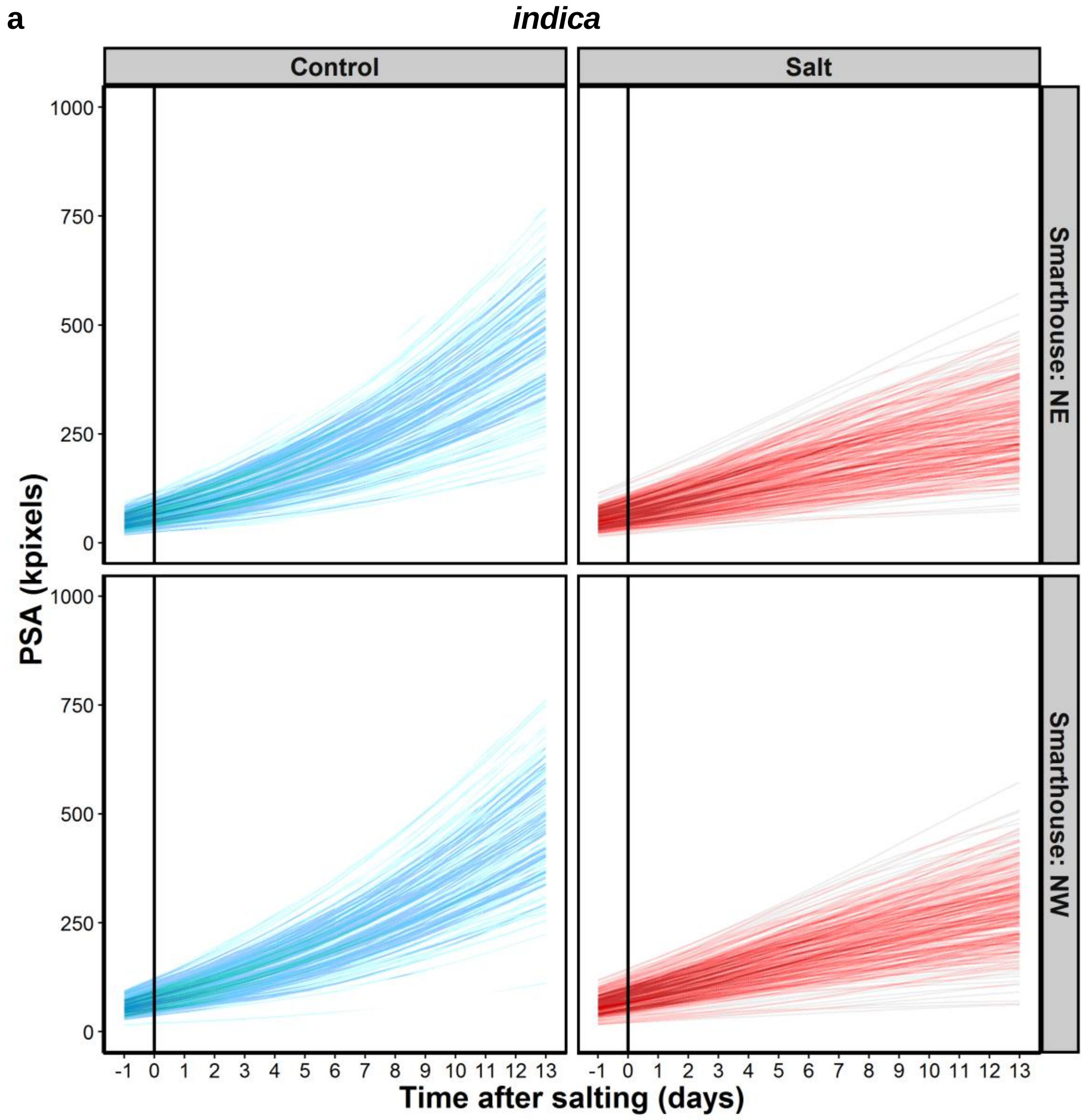
a
indica
Control
Salt
Smarthouse: NE
Smarthouse: NW
PSA (kpixels)
0
250
500
750
1000
-1 0 1 2 3 4 5 6 7 8 9 10 11 12 13
Time after salting (days)

b

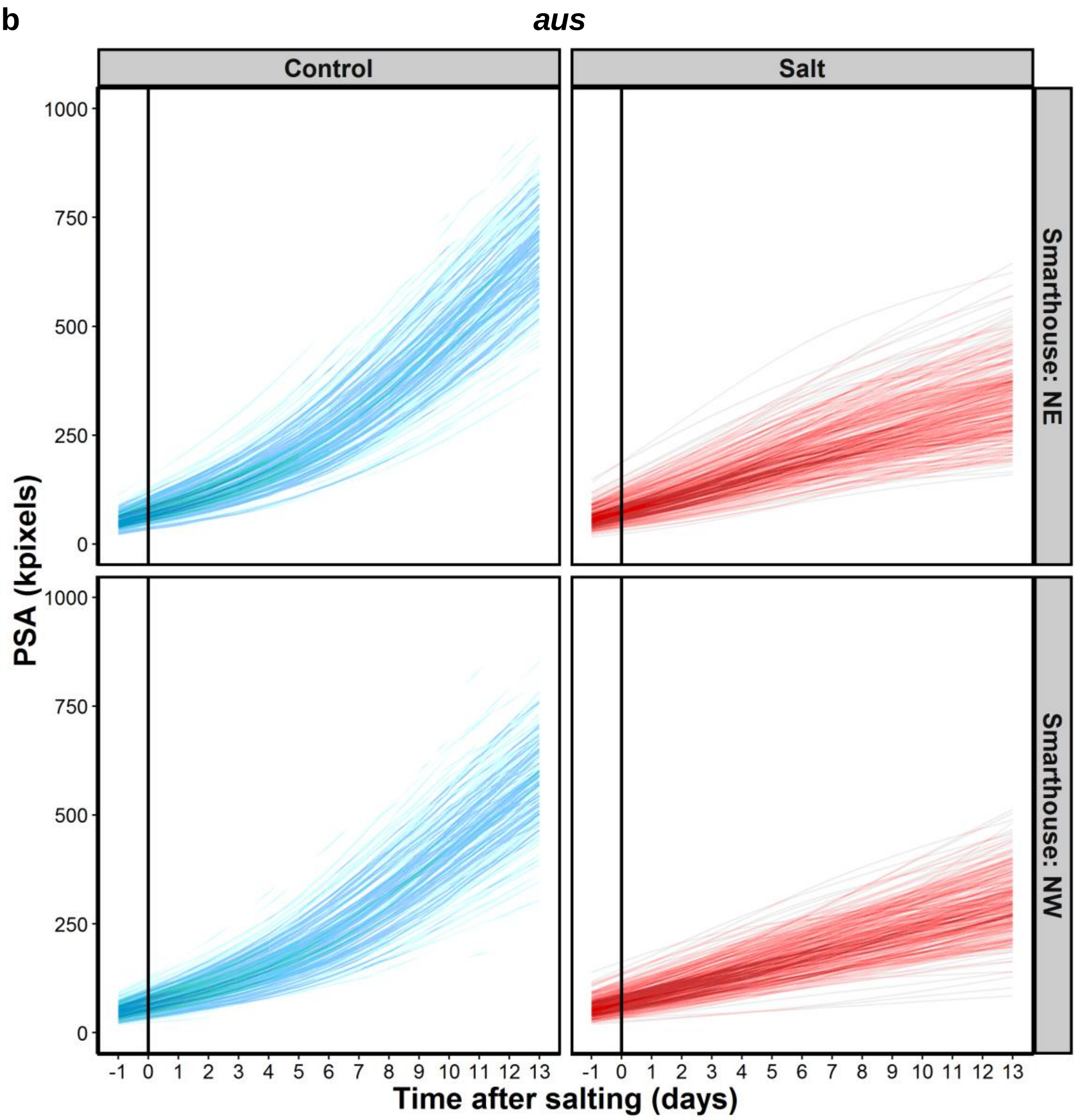
aus
Control
Salt
Smarthouse: NE
Smarthouse: NW
PSA (kpixels)
0
250
500
750
1000
-1 0 1 2 3 4 5 6 7 8 9 10 11 12 13
Time after salting (days)

c

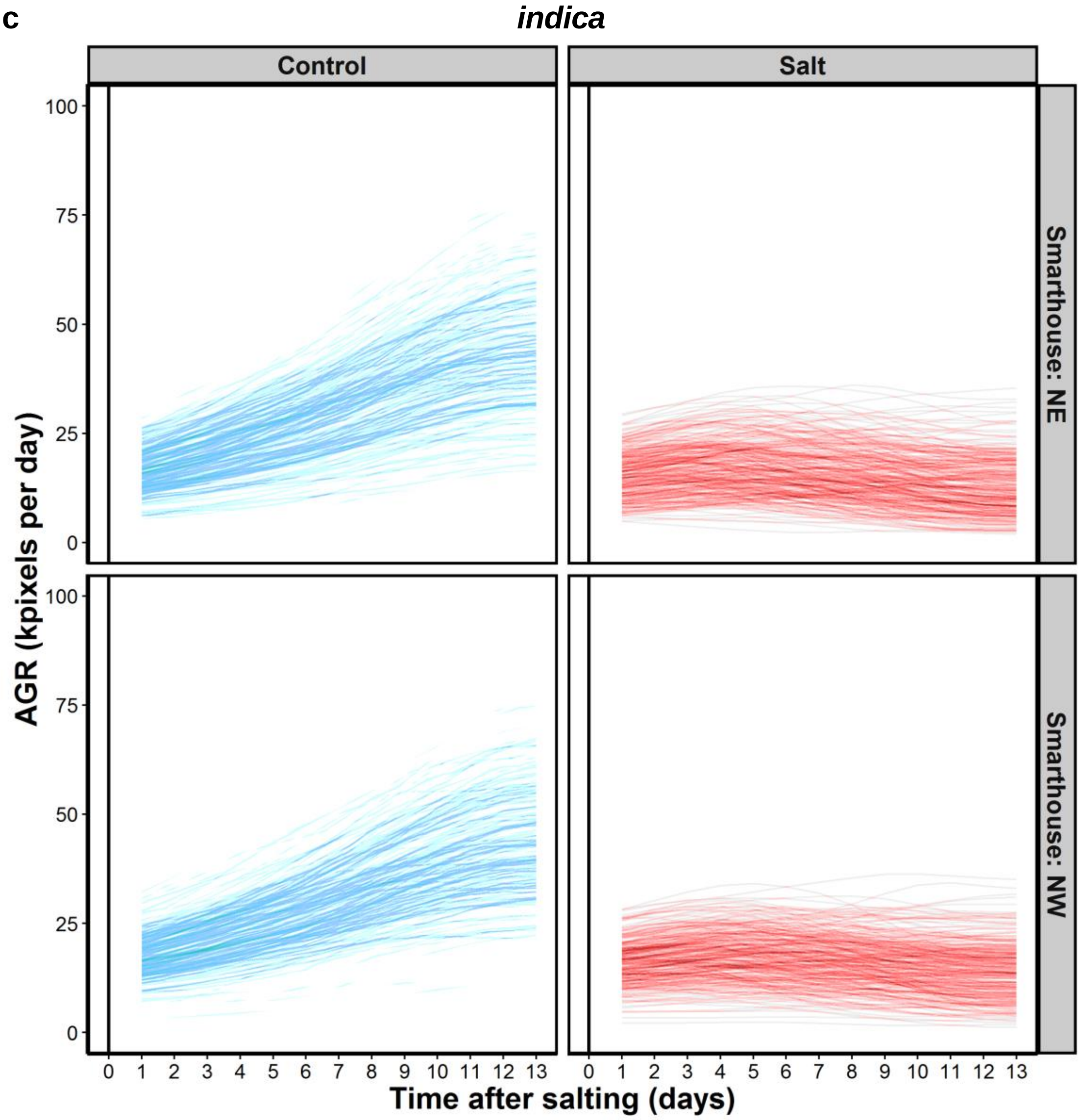
indica
Control
Salt
Smarthouse: NE
Smarthouse: NW
AGR (kpixels per day)
0
25
50
75
100
0 1 2 3 4 5 6 7 8 9 10 11 12 13
Time after salting (days)

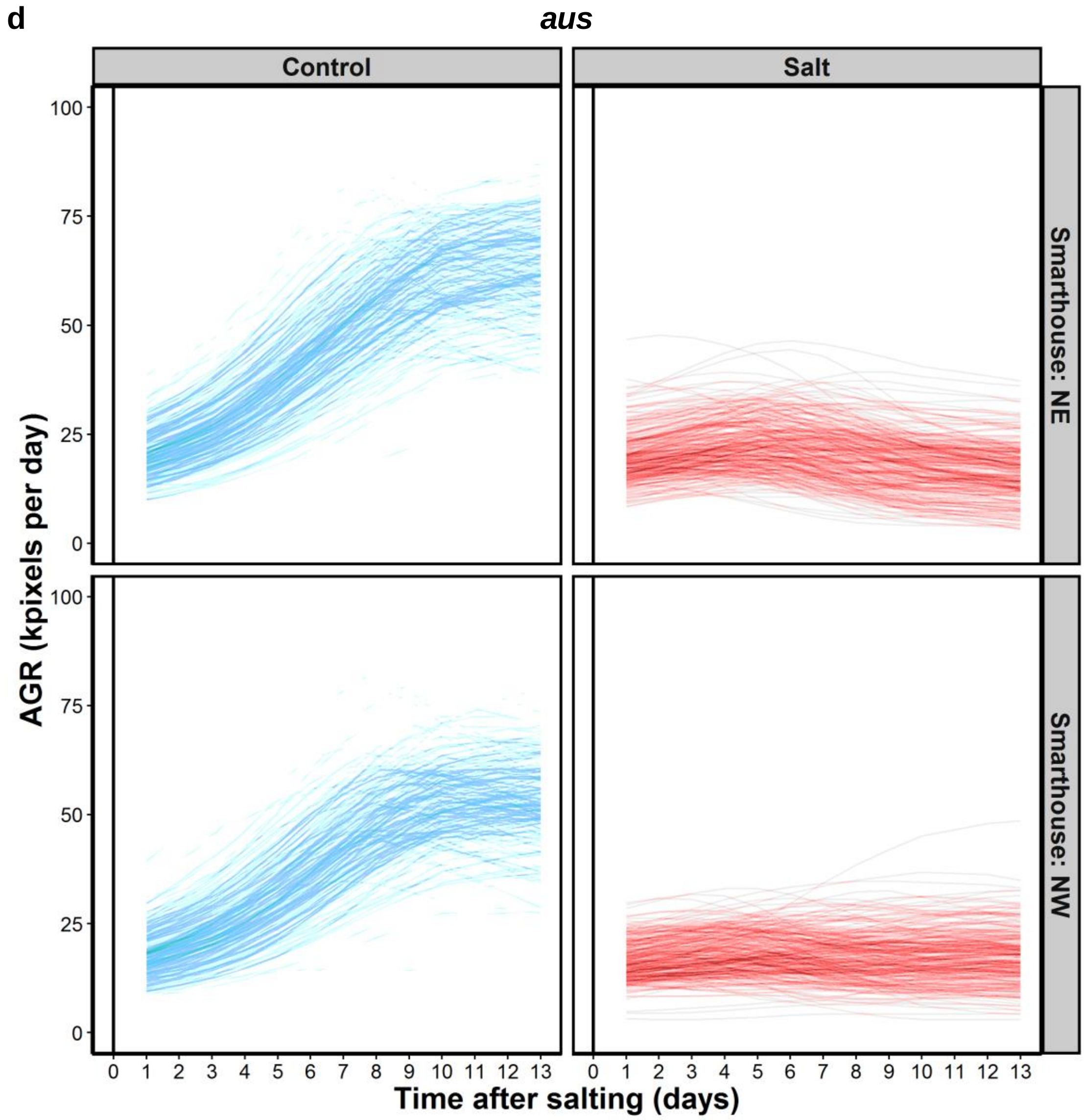

d
aus
Control
Salt
Smarthouse: NE
Smarthouse: NW
AGR (kpixels per day)
0
25
50
75
100
0 1 2 3 4 5 6 7 8 9 10 11 12 13
Time after salting (days)

e

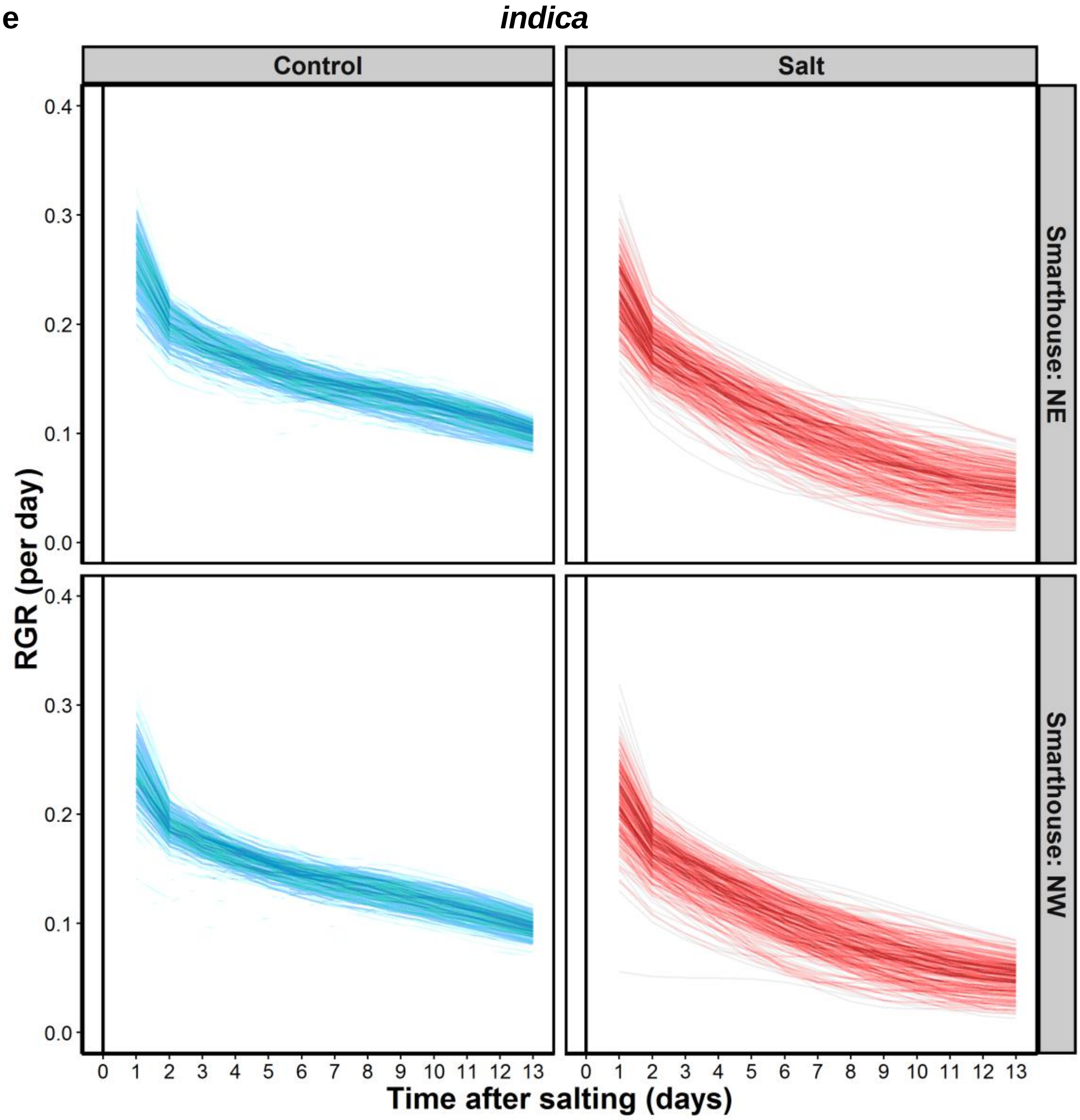
indica
Control
Salt
Smarthouse: NE
Smarthouse: NW
RGR (per day)
0.0
0.1
0.2
0.3
0.4
0 1 2 3 4 5 6 7 8 9 10 11 12 13
Time after salting (days)

f

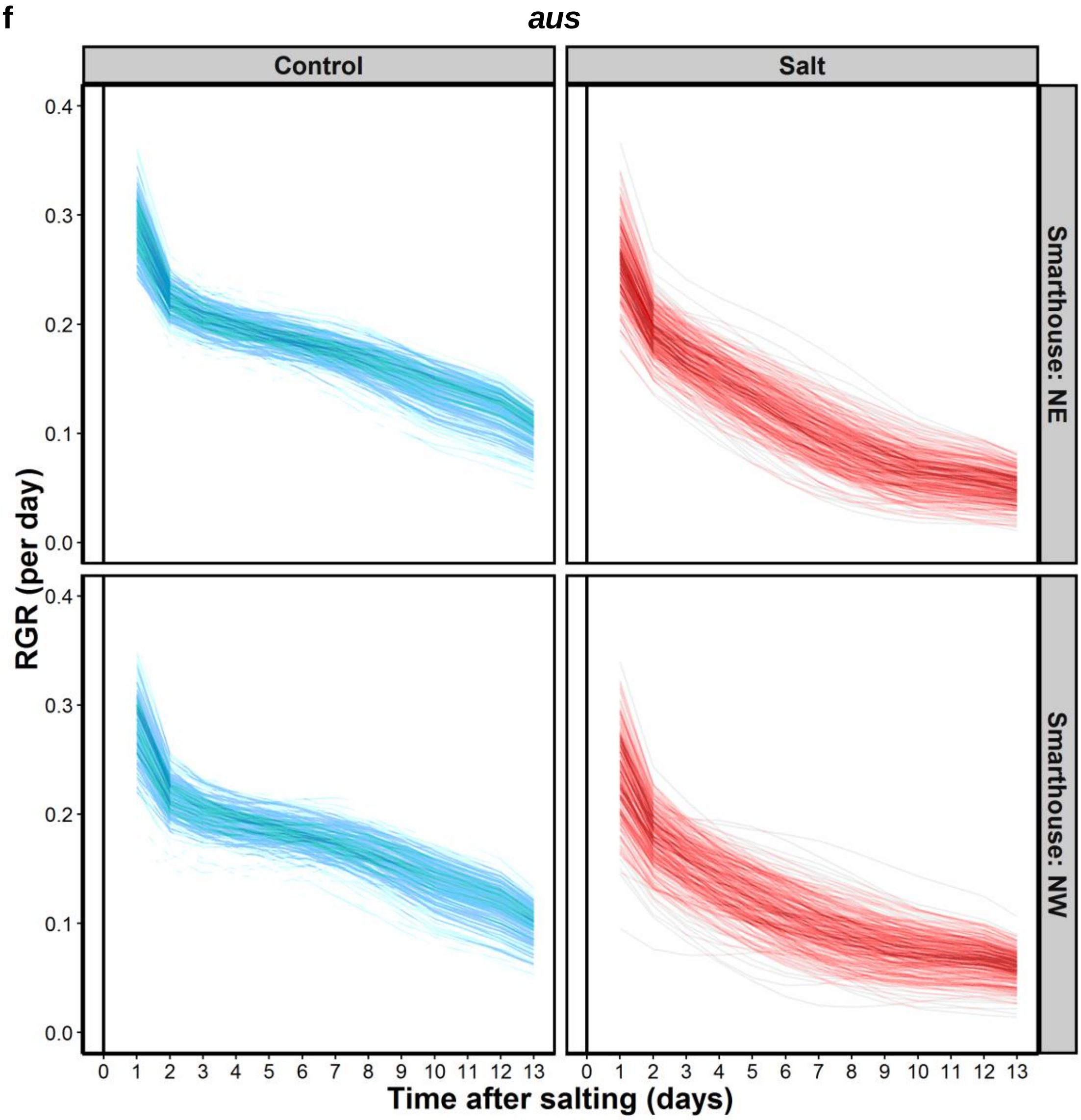
aus
Control
Salt
Smarthouse: NE
Smarthouse: NW
RGR (per day)
0.0
0.1
0.2
0.3
0.4
0 1 2 3 4 5 6 7 8 9 10 11 12 13
Time after salting (days)

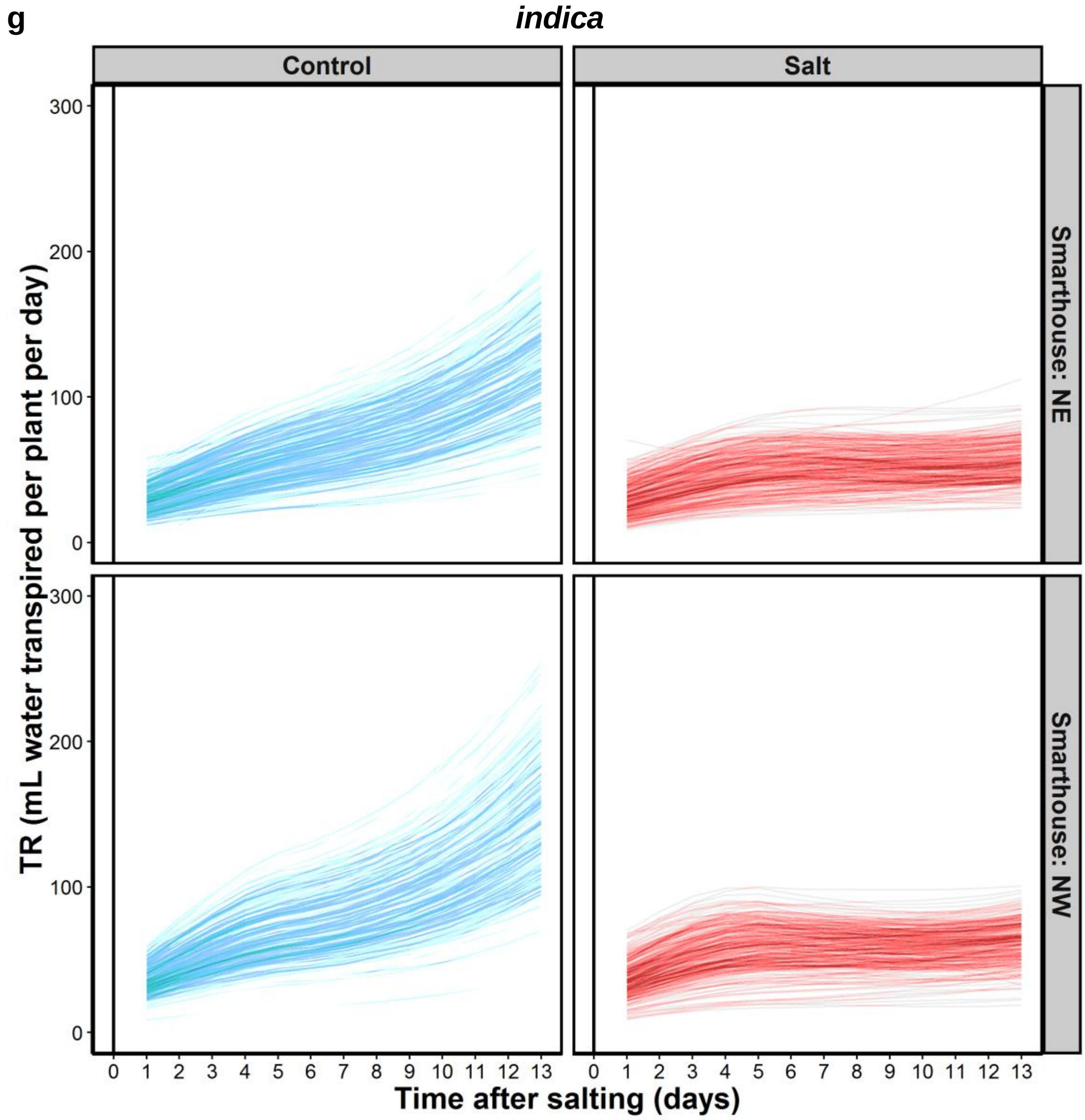

g
indica
Control
Salt
Smarthouse: NE
Smarthouse: NW
TR (mL water transpired per plant per day)
300
200
100
0
0 1 2 3 4 5 6 7 8 9 10 11 12 13
Time after salting (days)

h

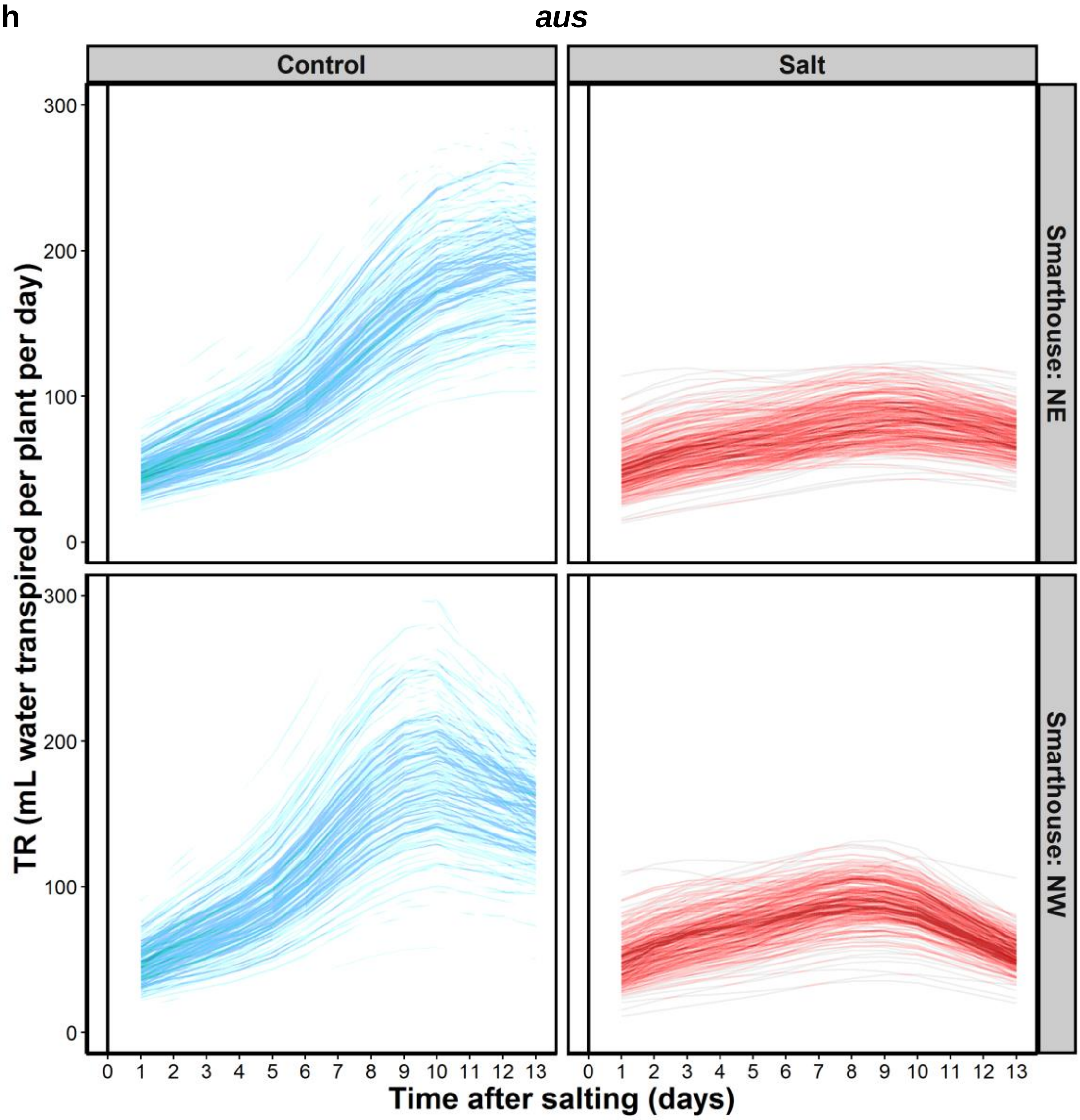
aus
Control
Salt
Smarthouse: NE
Smarthouse: NW
TR (mL water transpired per plant per day)
0
100
200
300
0 1 2 3 4 5 6 7 8 9 10 11 12 13
Time after salting (days)

i

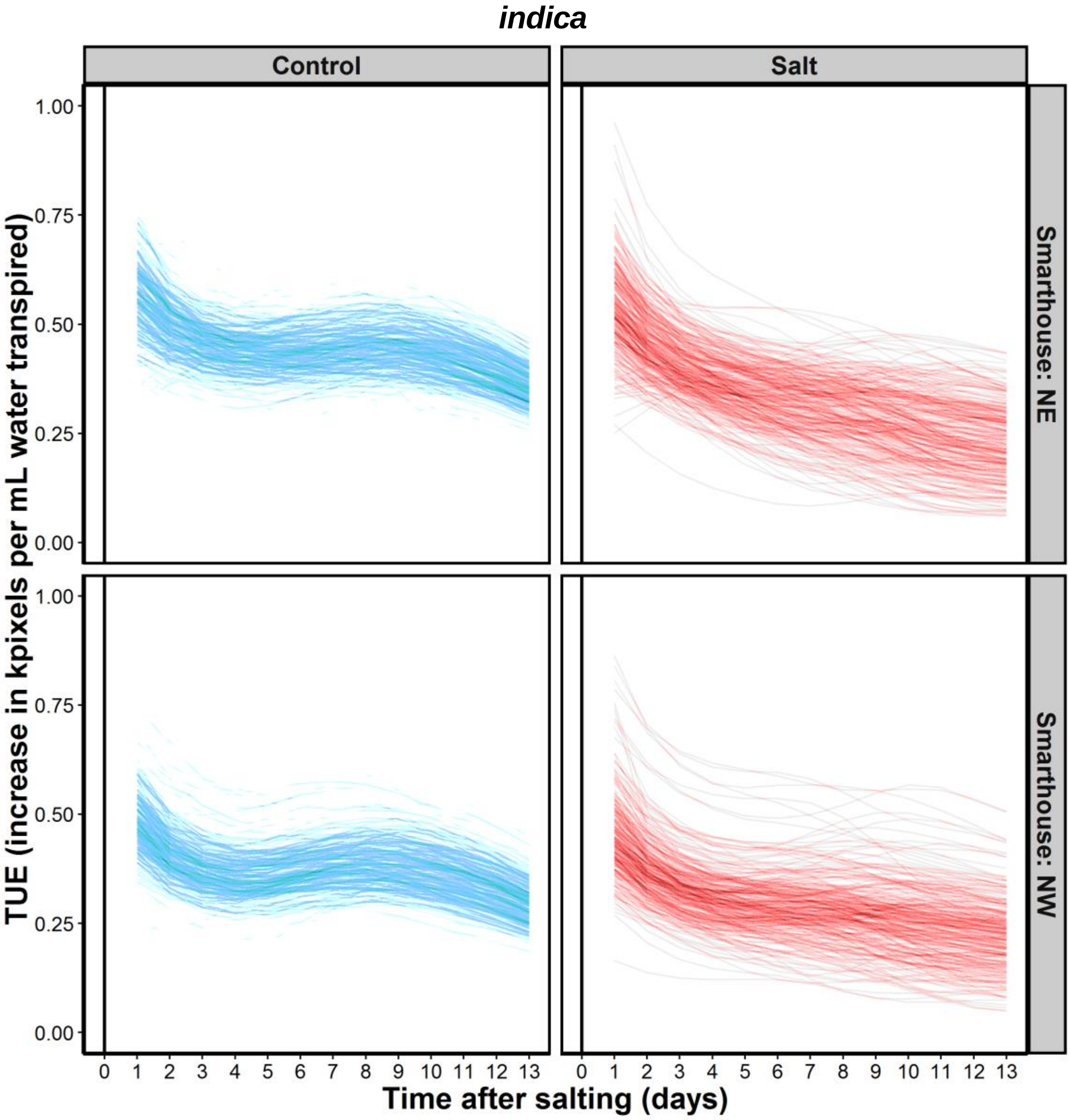

indica
Control
Salt
Smarthouse: NE
Smarthouse: NW
TUE (increase in kpixels per mL water transpired)
1.00
0.75
0.50
0.25
0.00
0 1 2 3 4 5 6 7 8 9 10 11 12 13
Time after salting (days)

j

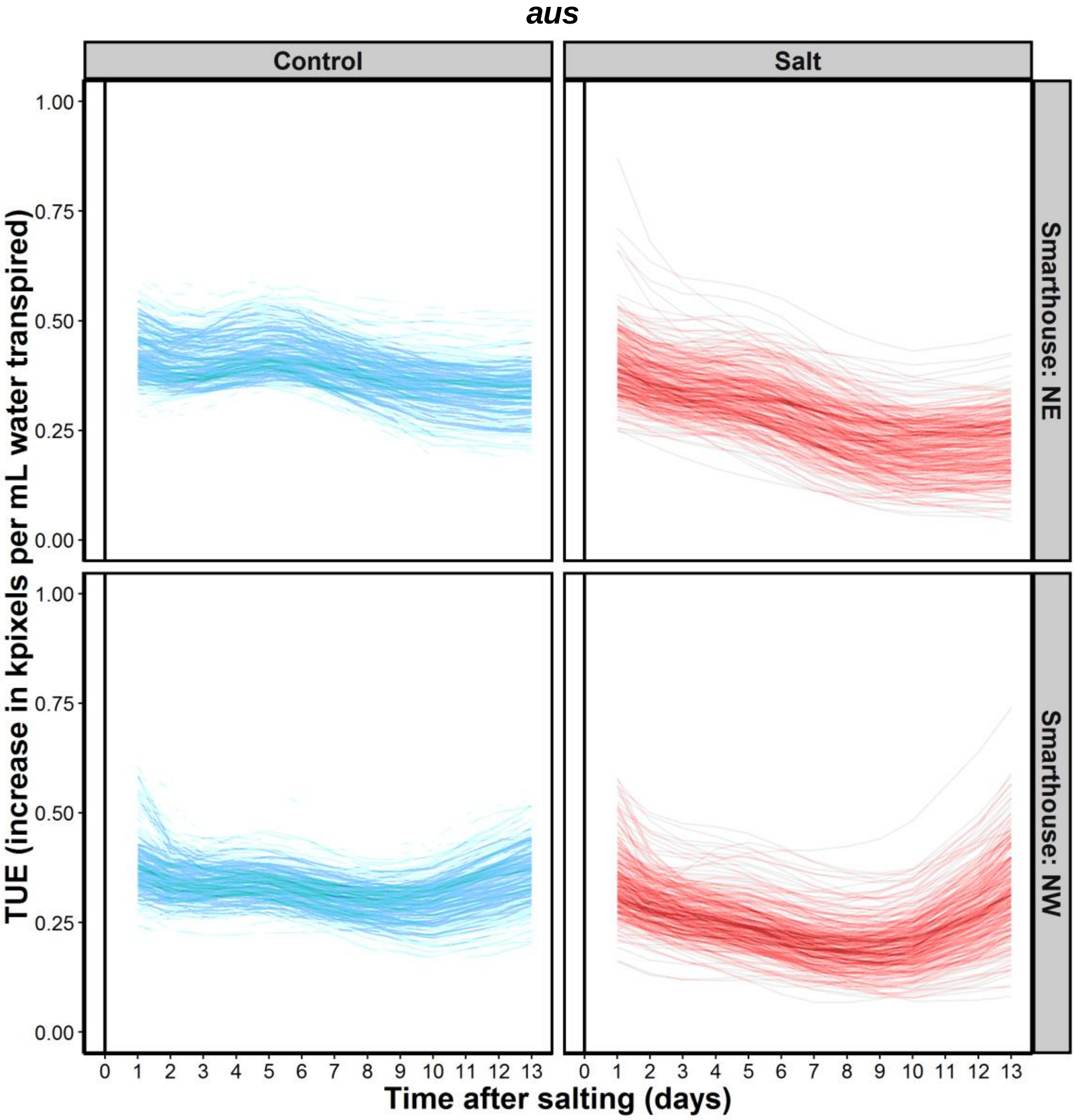
aus
Control
Salt
Smarthouse: NE
Smarthouse: NW
TUE (increase in kpixels per mL water transpired)
1.00
0.75
0.50
0.25
0.00
0 1 2 3 4 5 6 7 8 9 10 11 12 13
Time after salting (days)

k

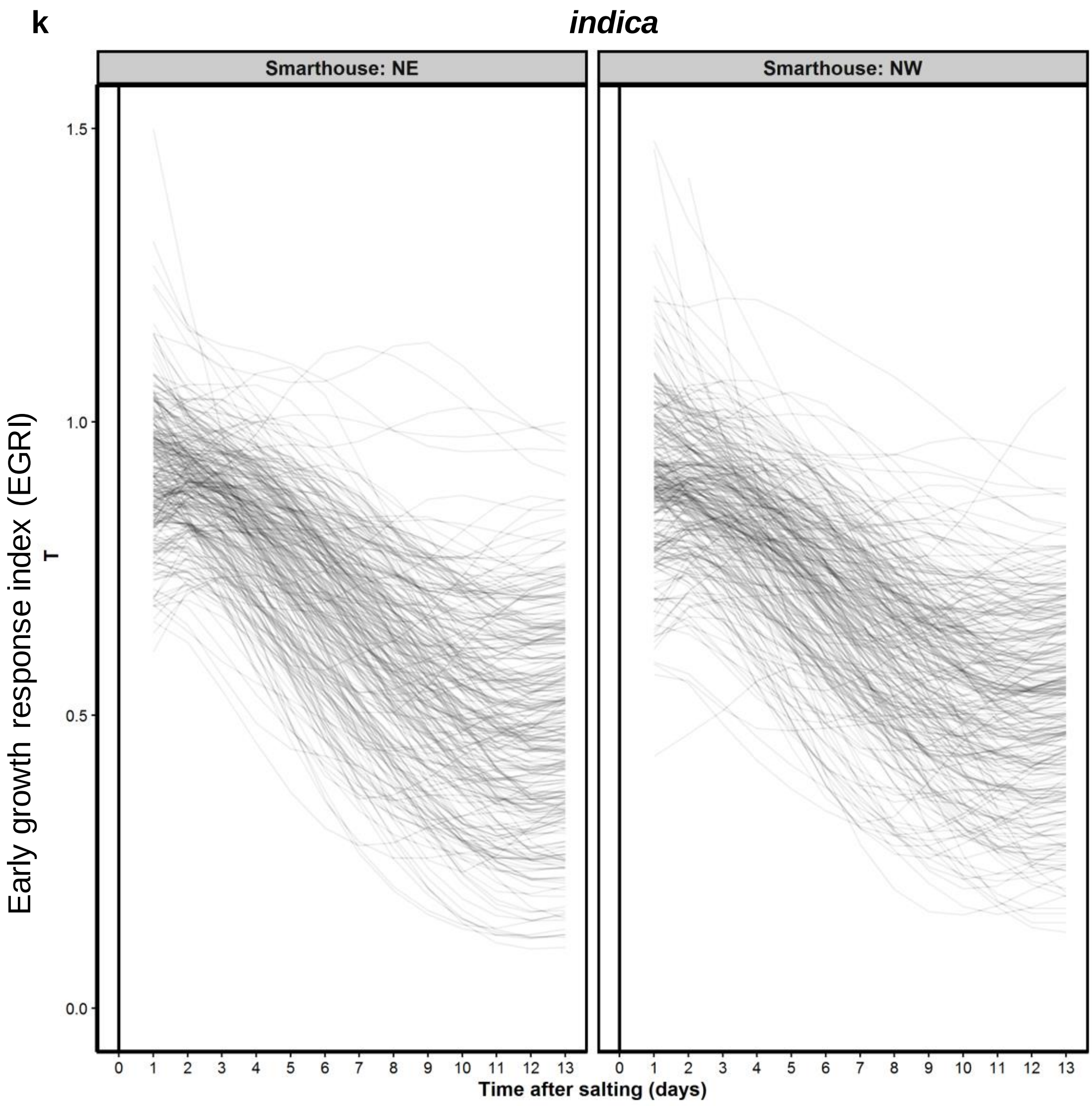
indica
Smarthouse: NE
Smarthouse: NW
Early growth response index (EGRI)
T
1.5
1.0
0.5
0.0
0 1 2 3 4 5 6 7 8 9 10 11 12 13
Time after salting (days)

l

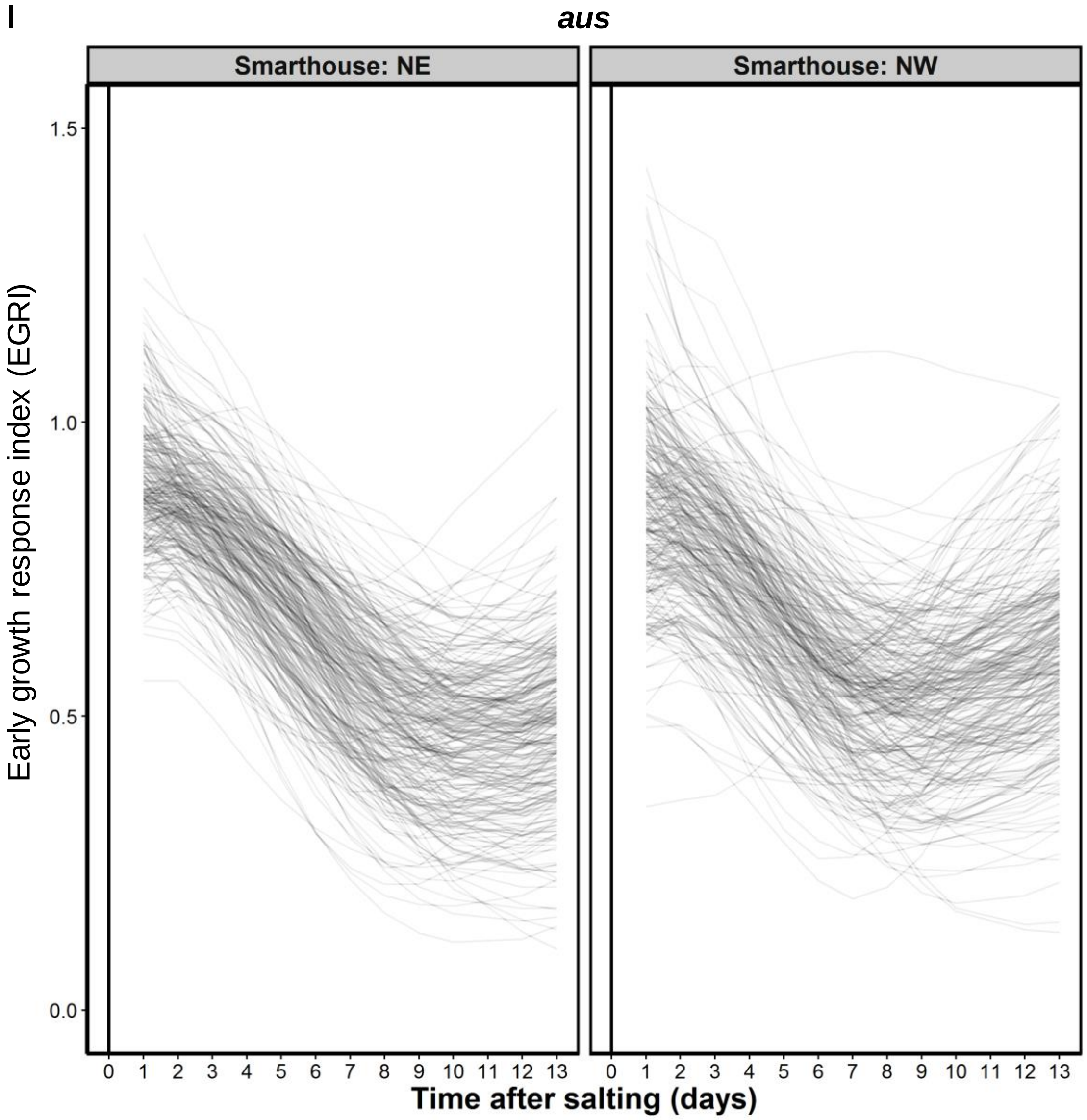

Supplementary Figure 1: Cubic spline lines for the values of each parameter for each individual plant for *indica* and *aus* in each Smarthouse.

(a) Splines of projected shoot area (PSA) for each individual plant of the *indica* panel.(**b**) As in **a**, with the *aus* panel. (**c**) Splines of absolute growth rate (AGR) for each individual plant of the *indica* panel. (**d**) As in **c**, with the *aus* panel. (**e**) Splines of relative growth rate (RGR) for each individual plant of the *indica* panel. (**f**) As in **e**, with the *aus* panel. (**g**) Splines of transpiration rate (TR) for each individual plant of the *indica* panel. (**h**) As in **g**, with the *aus* panel. (**i**) Splines of transpiration use efficiency (TUE) for each individual plant of the *indica* panel. (**j**) As **i**, with the *aus* panel. Lines represent control conditions (blue) and saline conditions (red) for each

plant (**k**) **S**plines of early growth response index (EGRI) index for each individual plant of the *indica* panel. (**l**) As in **k**, with the *aus* panel.

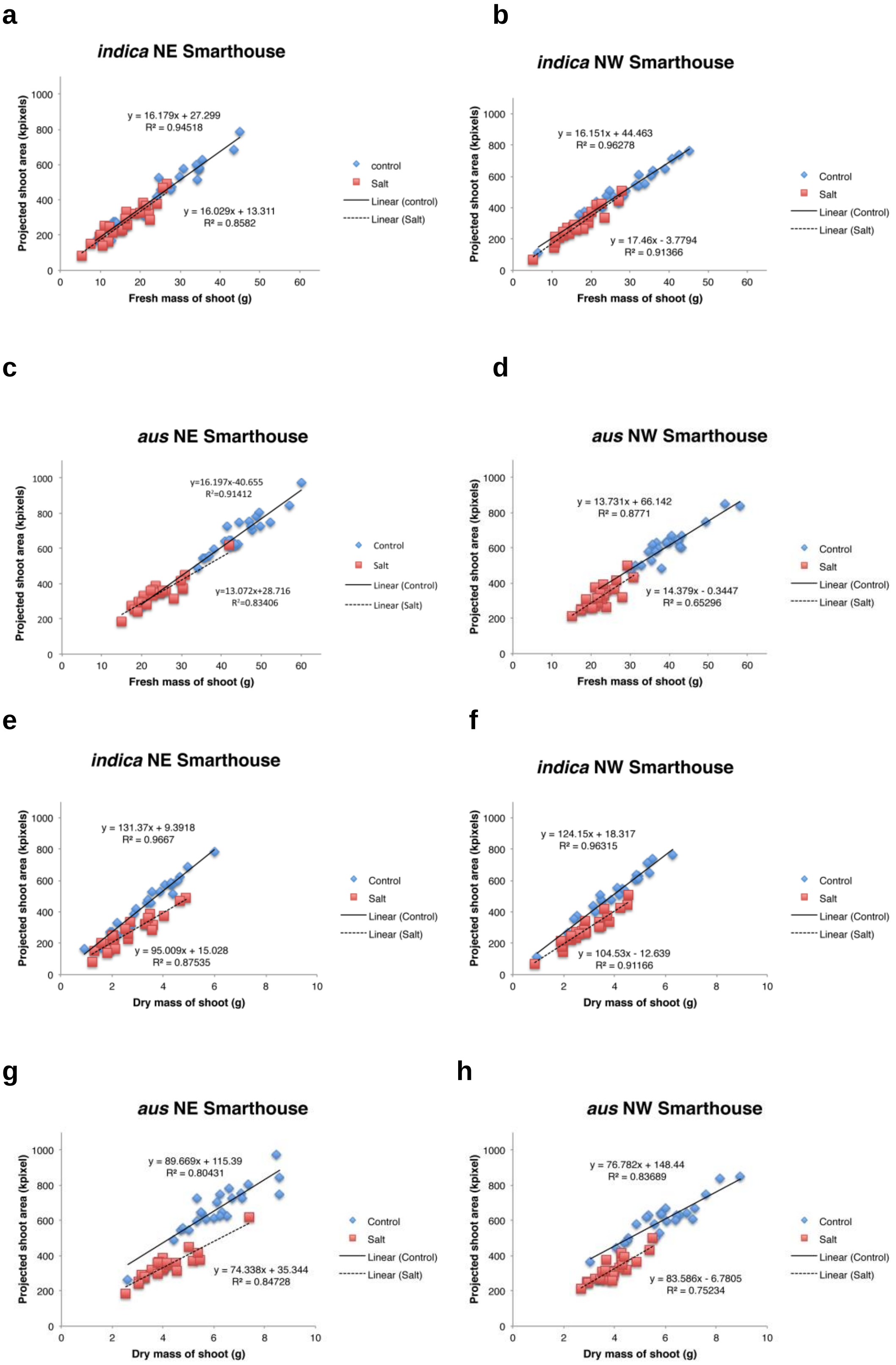
a
indica NE Smarthouse
y = 16.179x + 27.299
R² = 0.94518
y = 16.029x + 13.311
R² = 0.8582
control
Salt
Linear (control)
Linear (Salt)
Projected shoot area (kpixels)
Fresh mass of shoot (g)
b
indica NW Smarthouse
y = 16.151x + 44.463
R² = 0.96278
y = 17.46x - 3.7794
R² = 0.91366
Control
Salt
Linear (Control)
Linear (Salt)
Projected shoot area (kpixels)
Fresh mass of shoot (g)
c
aus NE Smarthouse
y=16.197x-40.655
R²=0.91412
y=13.072x+28.716
R²=0.83406
Control
Salt
Linear (Control)
Linear (Salt)
Projected shoot area (kpixels)
Fresh mass of shoot (g)
d
aus NW Smarthouse
y = 13.731x + 66.142
R² = 0.8771
y = 14.379x - 0.3447
R² = 0.65296
Control
Salt
Linear (Control)
Linear (Salt)
Projected shoot area (kpixels)
Fresh mass of shoot (g)
e
indica NE Smarthouse
y = 131.37x + 9.3918
R² = 0.9667
y = 95.009x + 15.028
R² = 0.87535
Control
Salt
Linear (Control)
Linear (Salt)
Projected shoot area (kpixels)
Dry mass of shoot (g)
f
indica NW Smarthouse
y = 124.15x + 18.317
R² = 0.96315
y = 104.53x - 12.639
R² = 0.91166
Control
Salt
Linear (Control)
Linear (Salt)
Projected shoot area (kpixels)
Dry mass of shoot (g)
g
aus NE Smarthouse
y = 89.669x + 115.39
R² = 0.80431
y = 74.338x + 35.344
R² = 0.84728
Control
Salt
Linear (Control)
Linear (Salt)
Projected shoot area (kpixel)
Dry mass of shoot (g)
h
aus NW Smarthouse
y = 76.782x + 148.44
R² = 0.83689
y = 83.586x - 6.7805
R² = 0.75234
Control
Salt
Linear (Control)
Linear (Salt)
Projected shoot area (kpixel)
Dry mass of shoot (g)

Supplementary Figure 2: Scatter plots relating projected shoot area (PSA) to shoot fresh mass and shoot dry mass.

(**a-d**) 24 control treated plants (blue) and 24 salt treated plants (red) were harvested on the last day of the experiment from each rice panel, *indica* and *aus*, in each Smarthouse (NE and NW). A positive linear relationship between projected shoot area PSA (kpixel) and shoot fresh mass was observed in both rice panels in each Smarthouse. (**e-h**) As in **a-d**, with PSA related to shoot dry mass.

a

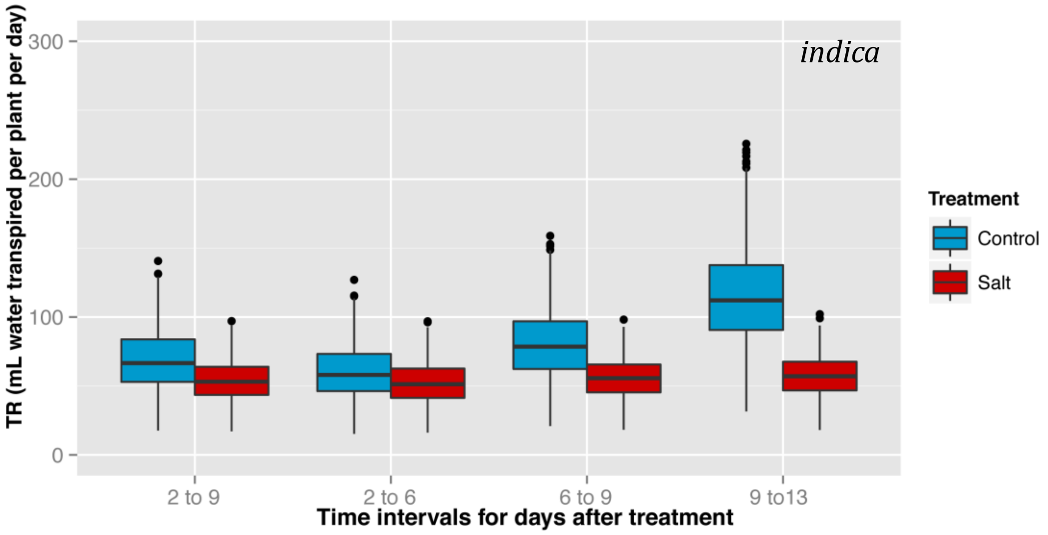
indica
TR (mL water transpired per plant per day)
300
200
100
0
2 to 9
2 to 6
6 to 9
9 to13
Time intervals for days after treatment
Treatment
Control
Salt

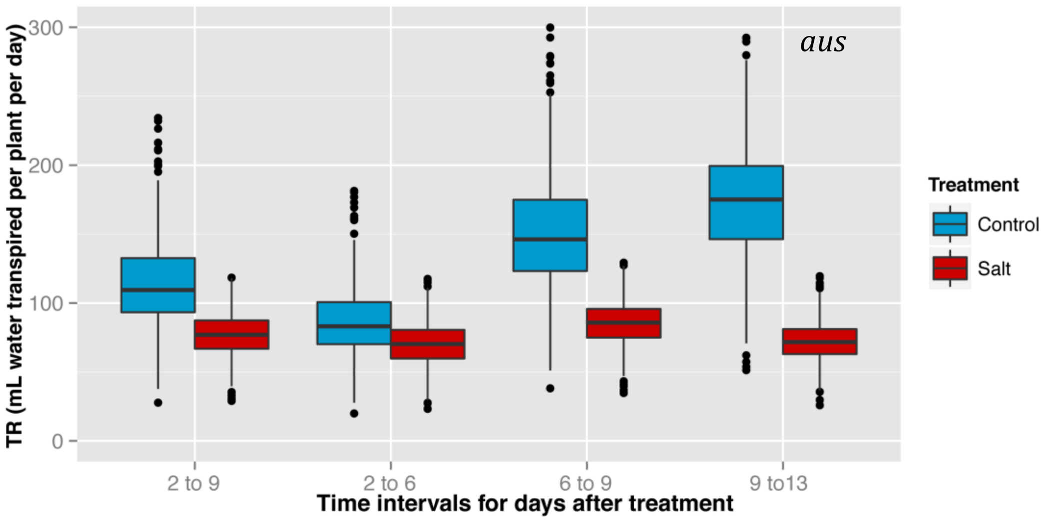
aus
TR (mL water transpired per plant per day)
300
200
100
0
2 to 9
2 to 6
6 to 9
9 to13
Time intervals for days after treatment
Treatment
Control
Salt

b

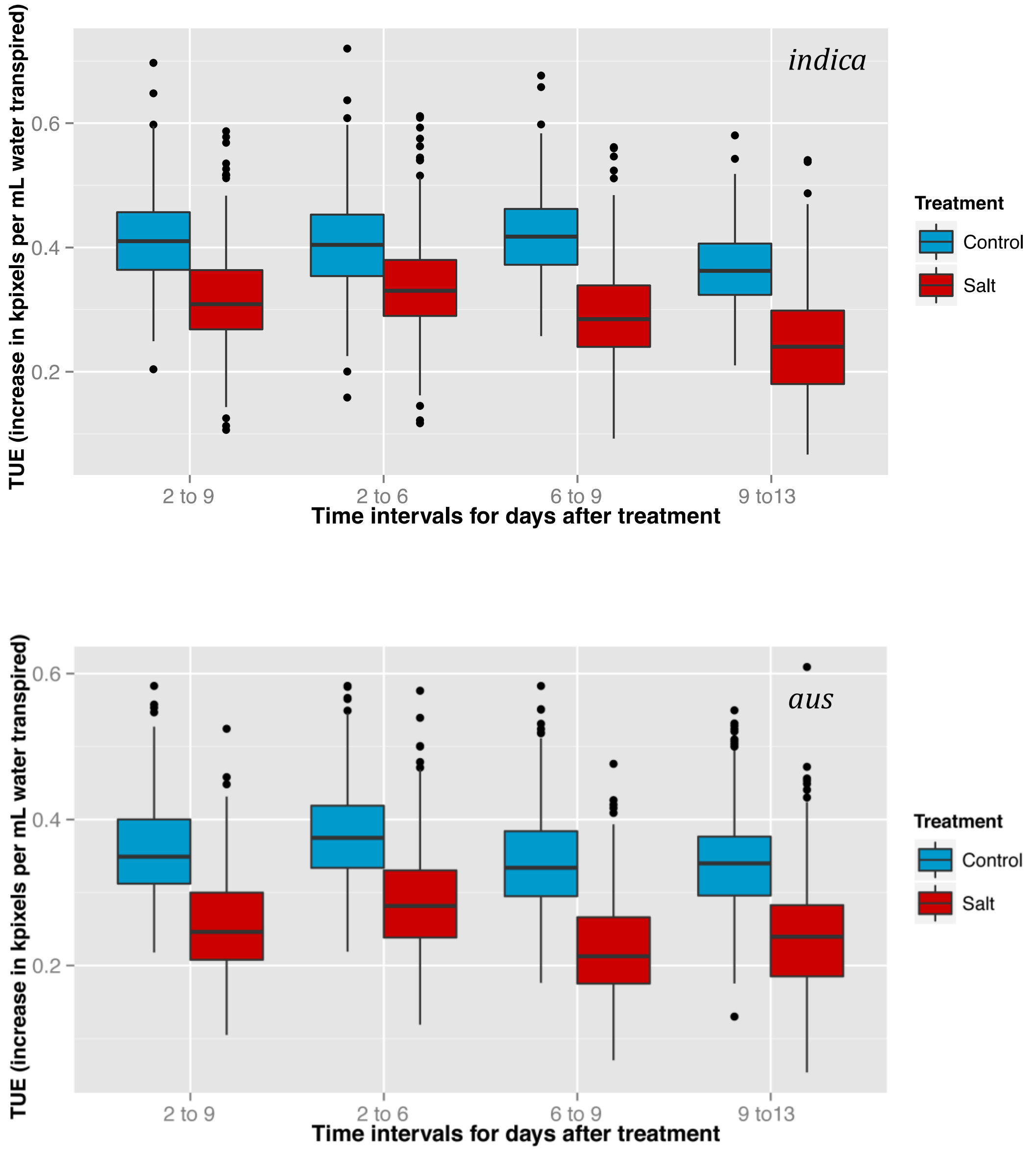

Supplementary Figure 3: Boxplots comparing TR and TUE of plants grown in control and saline conditions.

(**a**) Values of **transpiration rate** (TR) at different time intervals for *indica* (top) and *aus* (bottom).

(**b**) Values of **transpiration use efficiency** (TUE) at different time intervals for *indica* (top) and *aus* (bottom).

a ***indica***

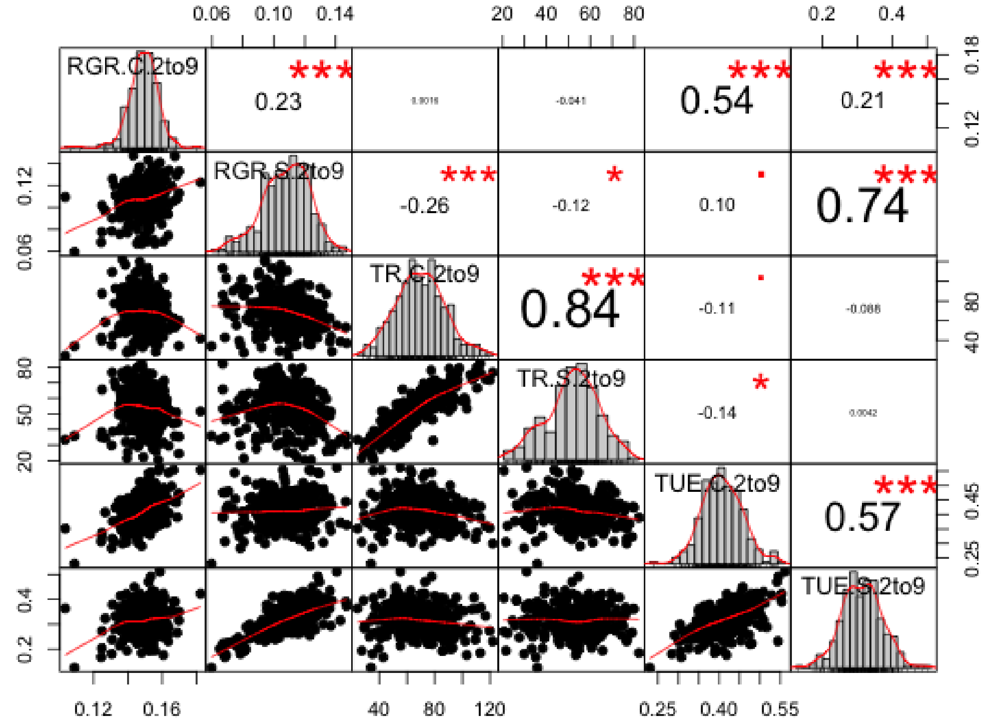

b ***indica***

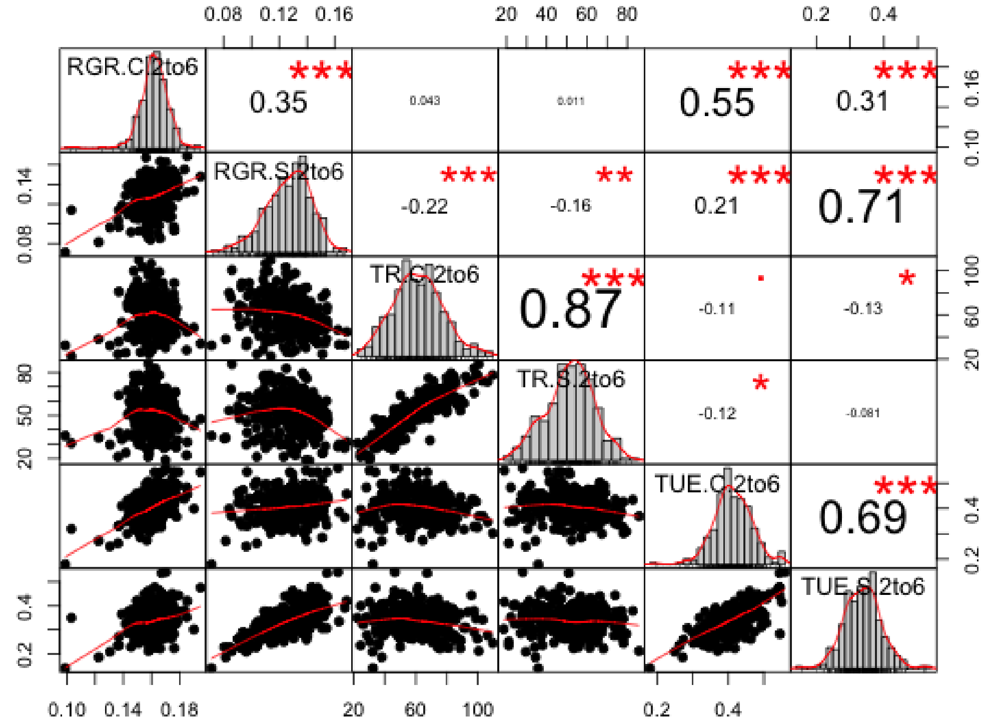

c *indica*

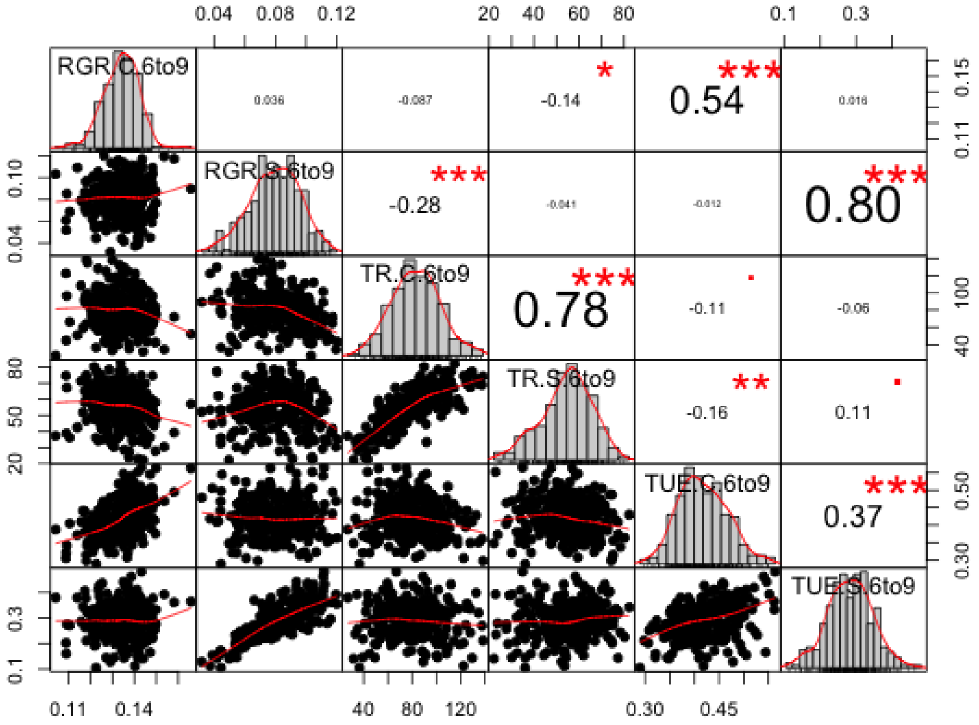

d *indica*

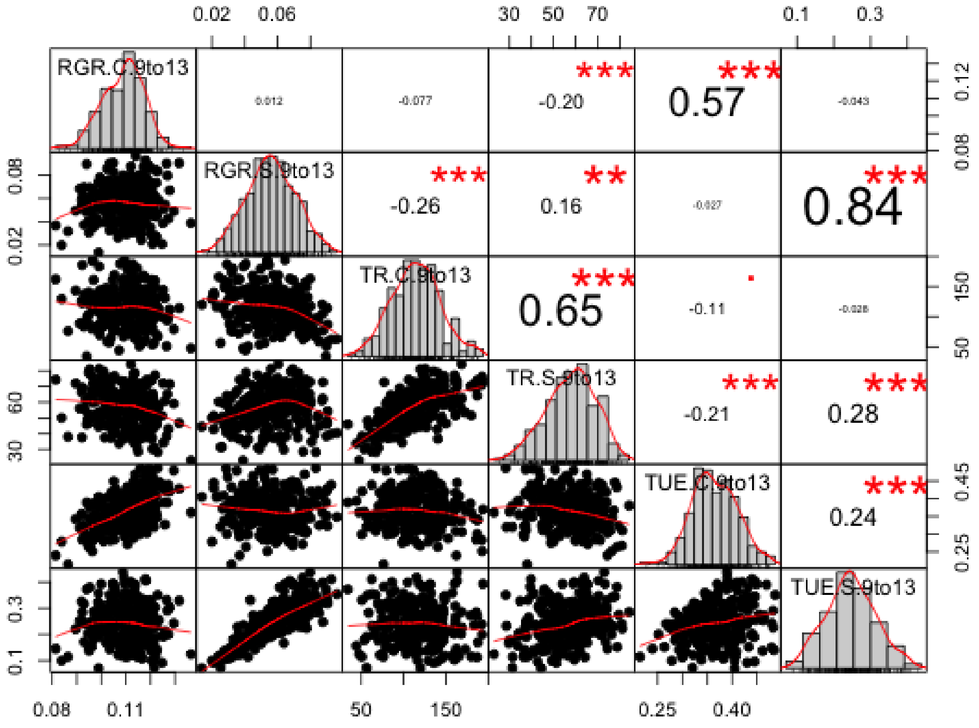

e *aus*

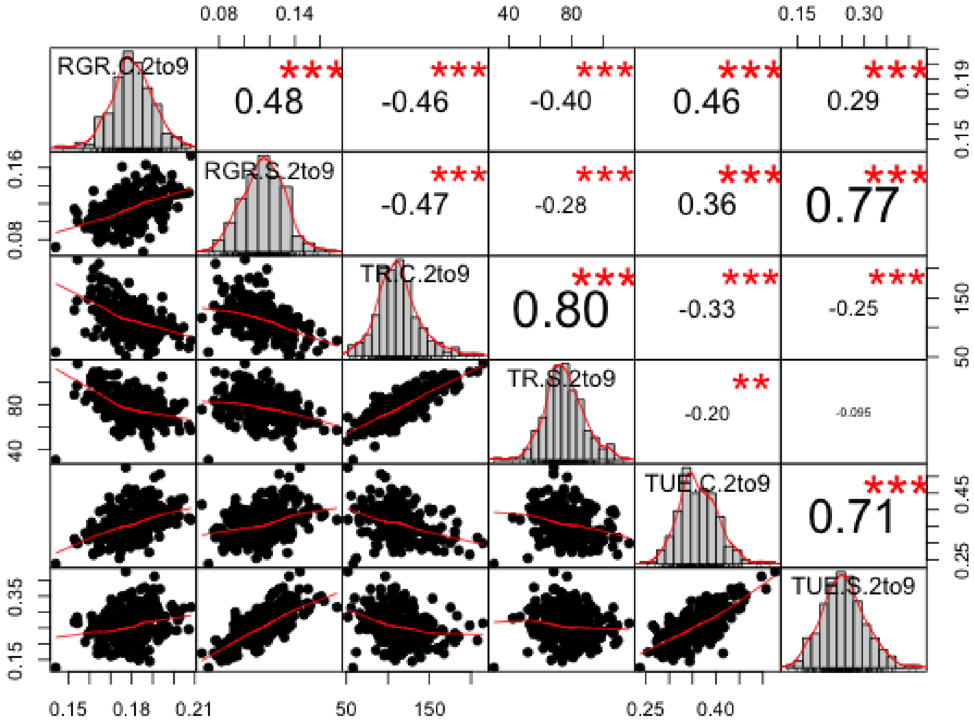
RGR.C.2to9
RGR.S.2to9
TR.C.2to9
TR.S.2to9
TUE.C.2to9
TUE.S.2to9
0.48
-0.46
-0.40
0.46
0.29
-0.47
-0.28
0.36
0.77
0.80
-0.33
-0.25
-0.20
-0.095
0.71
0.08 0.14
40 80
0.15 0.30
0.15 0.19
0.08 0.16
50 150
40 80
0.25 0.45
0.15 0.35
0.15 0.18 0.21
50 150
0.25 0.40

f *aus*

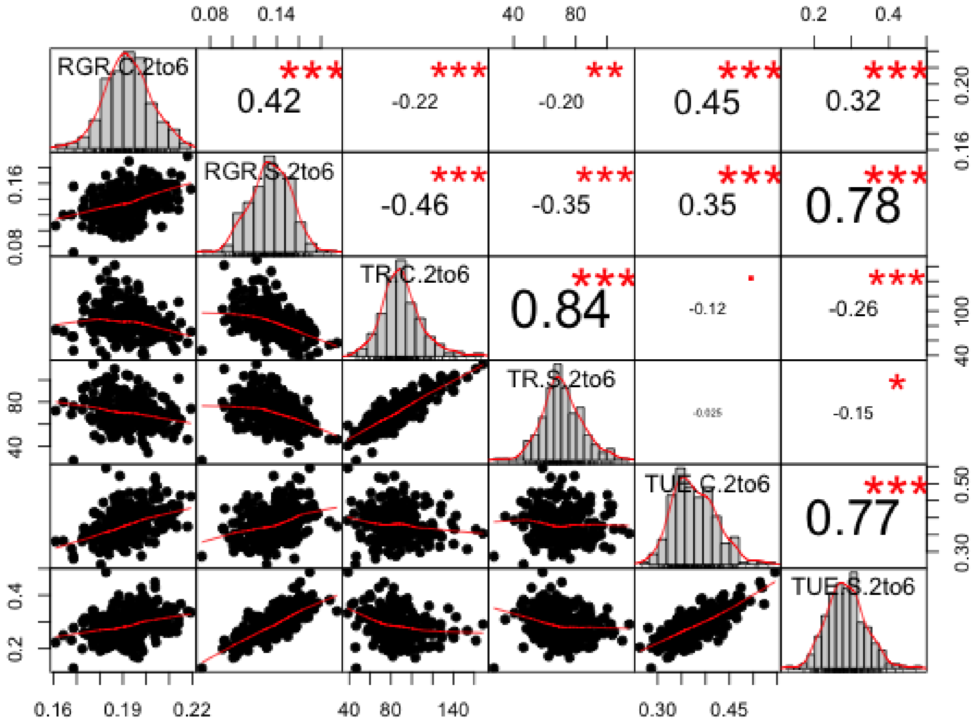
RGR.C.2to6
RGR.S.2to6
TR.C.2to6
TR.S.2to6
TUE.C.2to6
TUE.S.2to6
0.42
-0.22
-0.20
0.45
0.32
-0.46
-0.35
0.35
0.78
0.84
-0.12
-0.26
-0.025
-0.15
0.77
0.08 0.14
40 80
0.2 0.4
0.16 0.20
0.08 0.16
40 100
40 80
0.30 0.50
0.2 0.4
0.16 0.19 0.22
40 80 140
0.30 0.45

g ***aus***

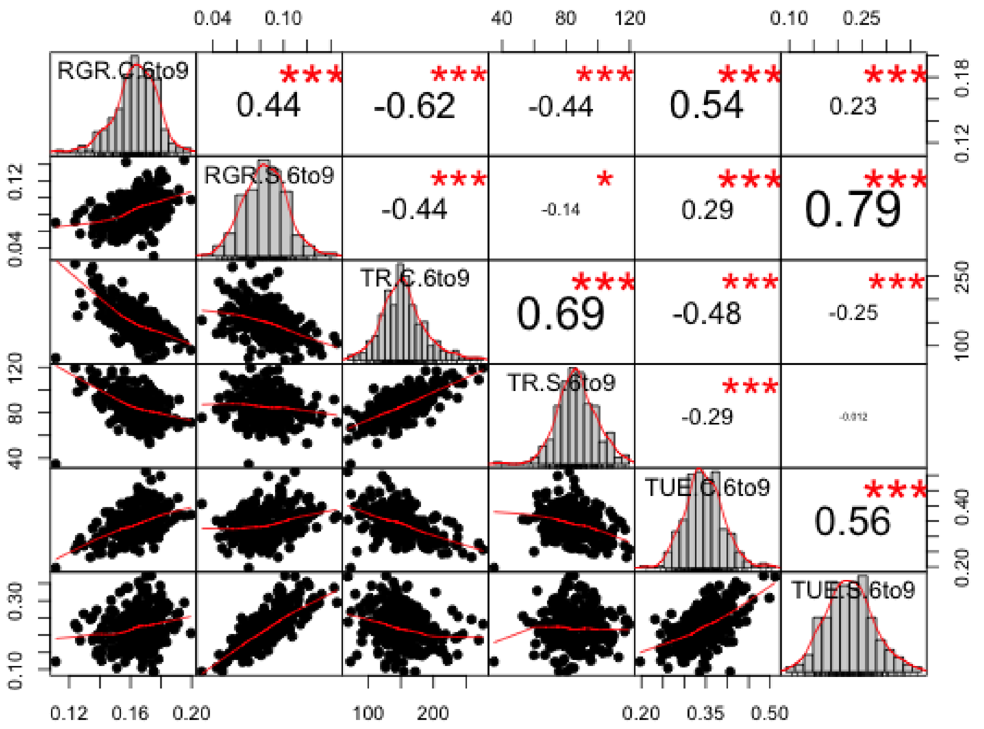
RGR.C.6to9
RGR.S.6to9
TR.C.6to9
TR.S.6to9
TUE.C.6to9
TUE.S.6to9
0.44
-0.62
-0.44
0.54
0.23
-0.44
-0.14
0.29
0.79
0.69
-0.48
-0.25
-0.29
-0.012
0.56

h ***aus***

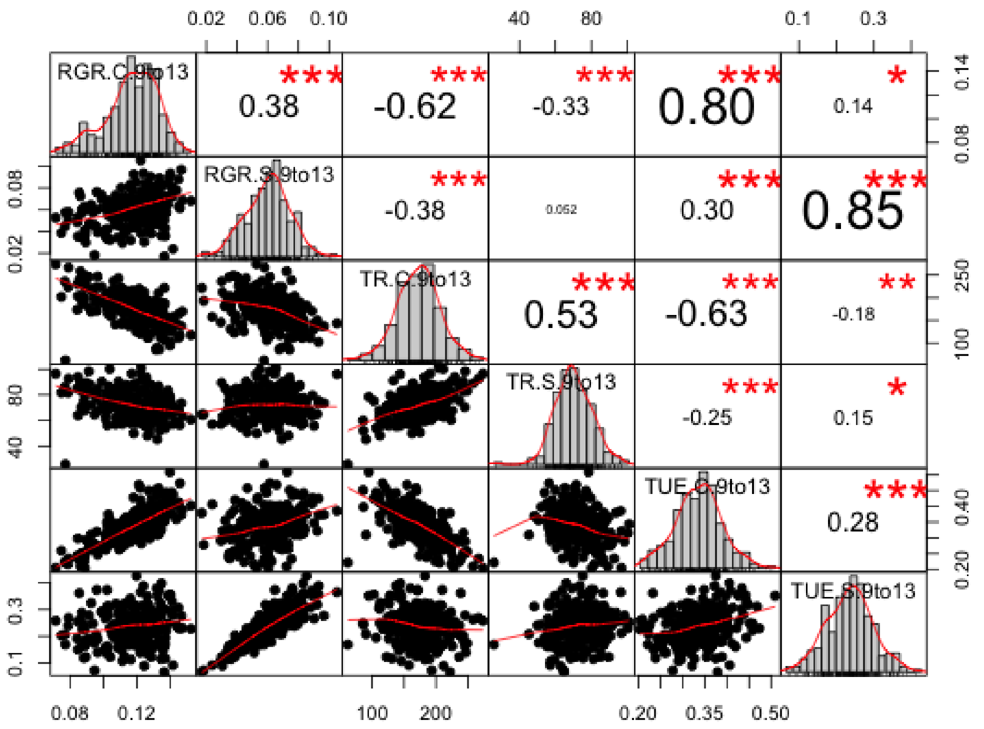
RGR.C.9to13
RGR.S.9to13
TR.C.9to13
TR.S.9to13
TUE.C.9to13
TUE.S.9to13
0.38
-0.62
-0.33
0.80
0.14
-0.38
0.052
0.30
0.85
0.53
-0.63
-0.18
-0.25
0.15
0.28

Supplementary Figure 4: Correlation matrices for *indica* and *aus*.

Pearson correlation analysis was conducted between the three main traits relative growth rate (RGR) (per day) transpiration rate (TR) (mL water transpired per plant per day) and transpiration use efficiency (TUE) (increase in kpixels per mL water transpired) at each time interval to assess the relationships between traits. The distribution of each variable is shown on the diagonal. The bottom half of the figure, below the diagonal, shows the bivariate scatter plots with a fitted line displayed. The top half of the figure, above the diagonal, displays the value of the correlation with the significance level shown with asterisks (*$P < 0.05$, **$P < 0.01$, ***$P < 0.001$).

a Model 'Marker' for relative growth rate (RGR)

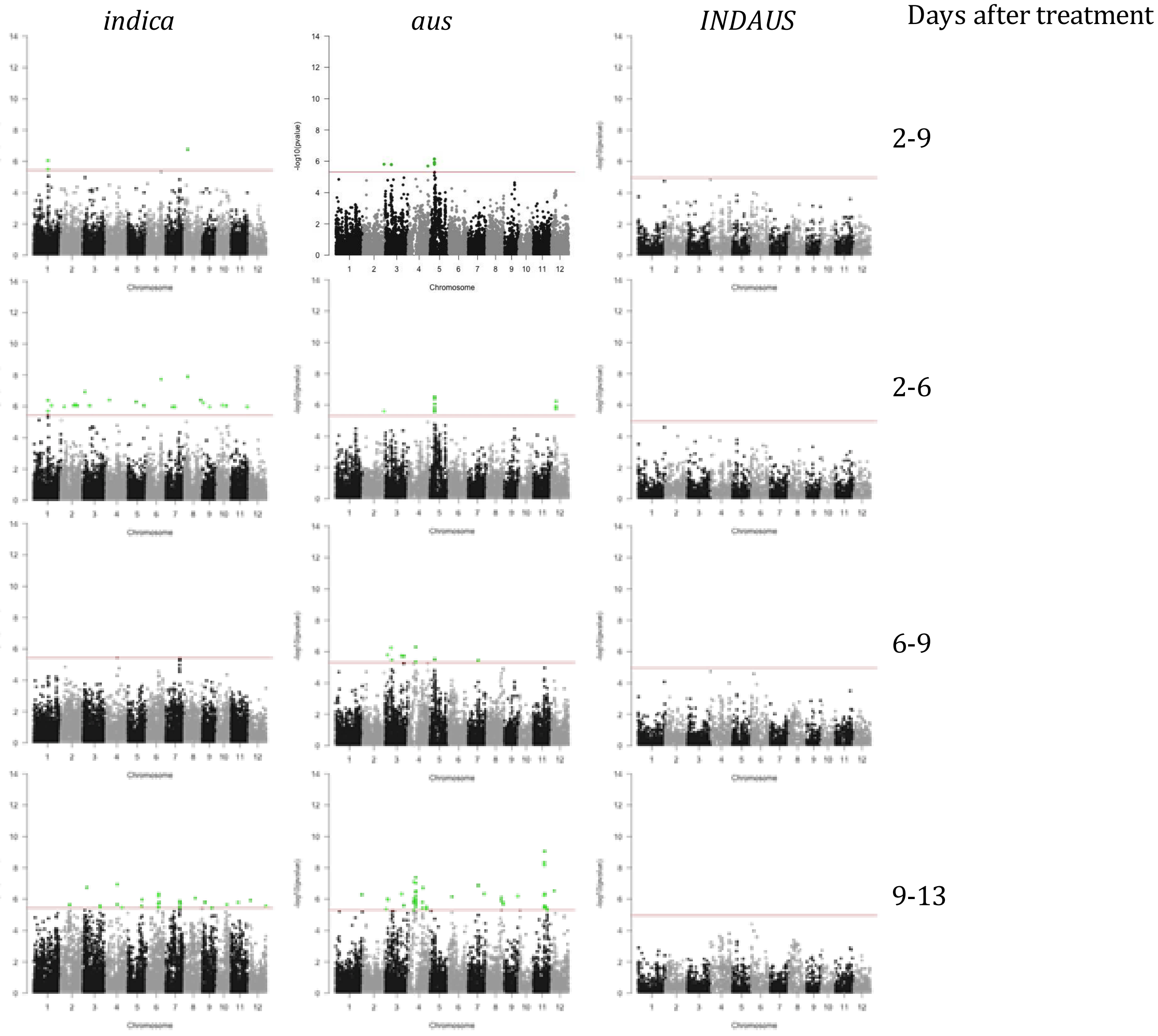

b

Model 'Marker' for transpiration rate (TR)

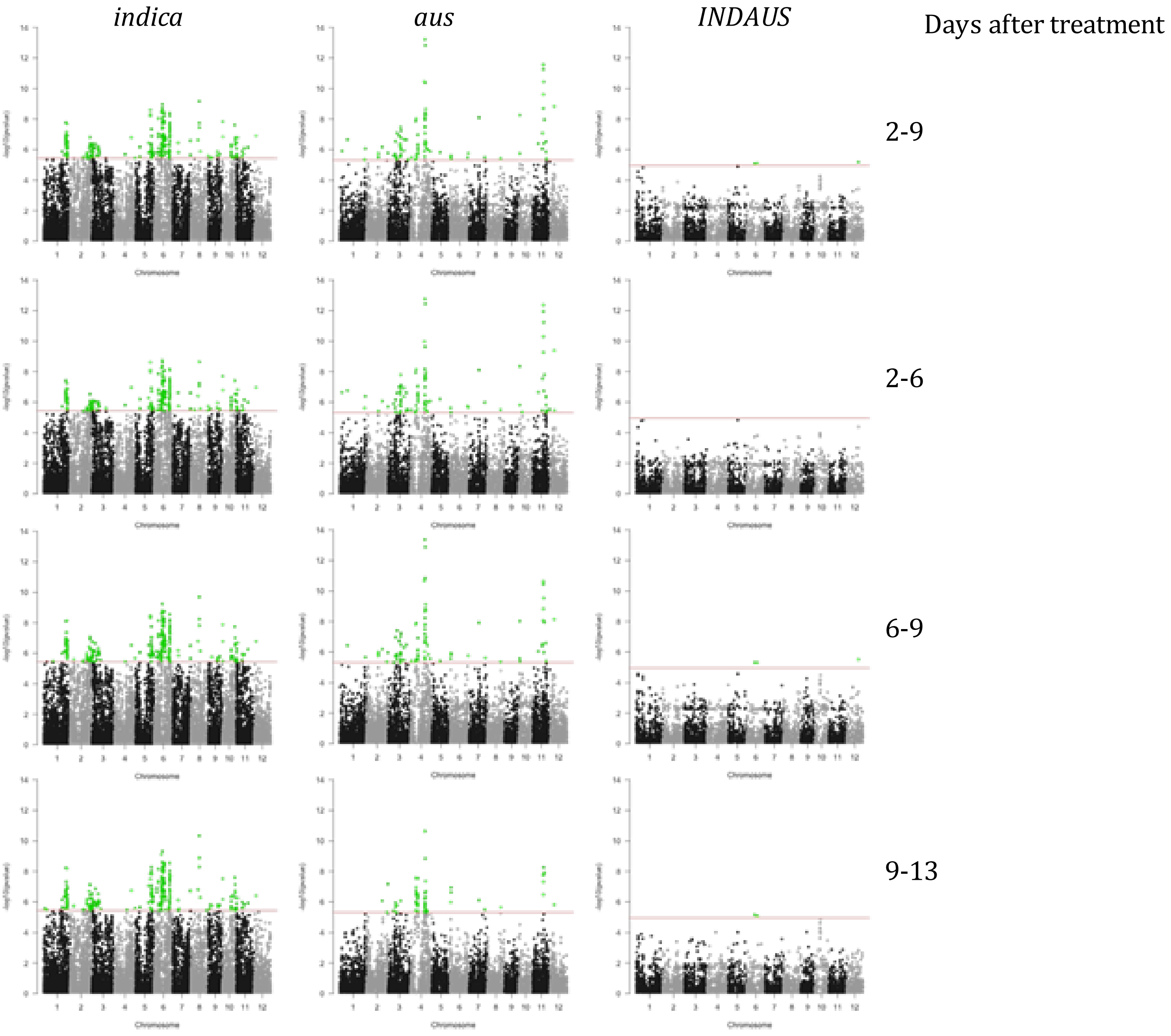

c Model 'Marker' for transpiration use efficiency (TUE)

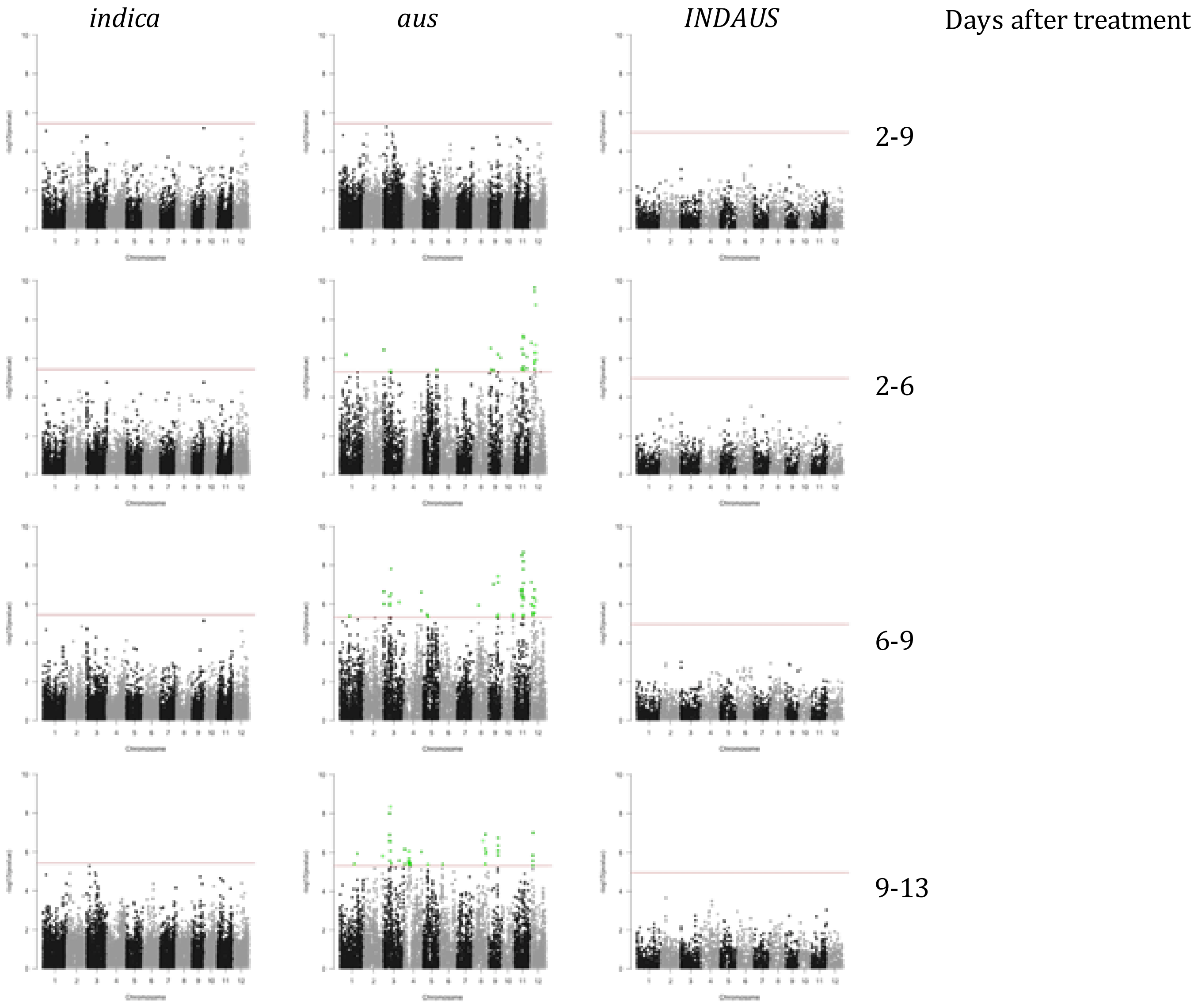

Model 'Marker-by-Treatment' for relative growth rate (RGR)

d

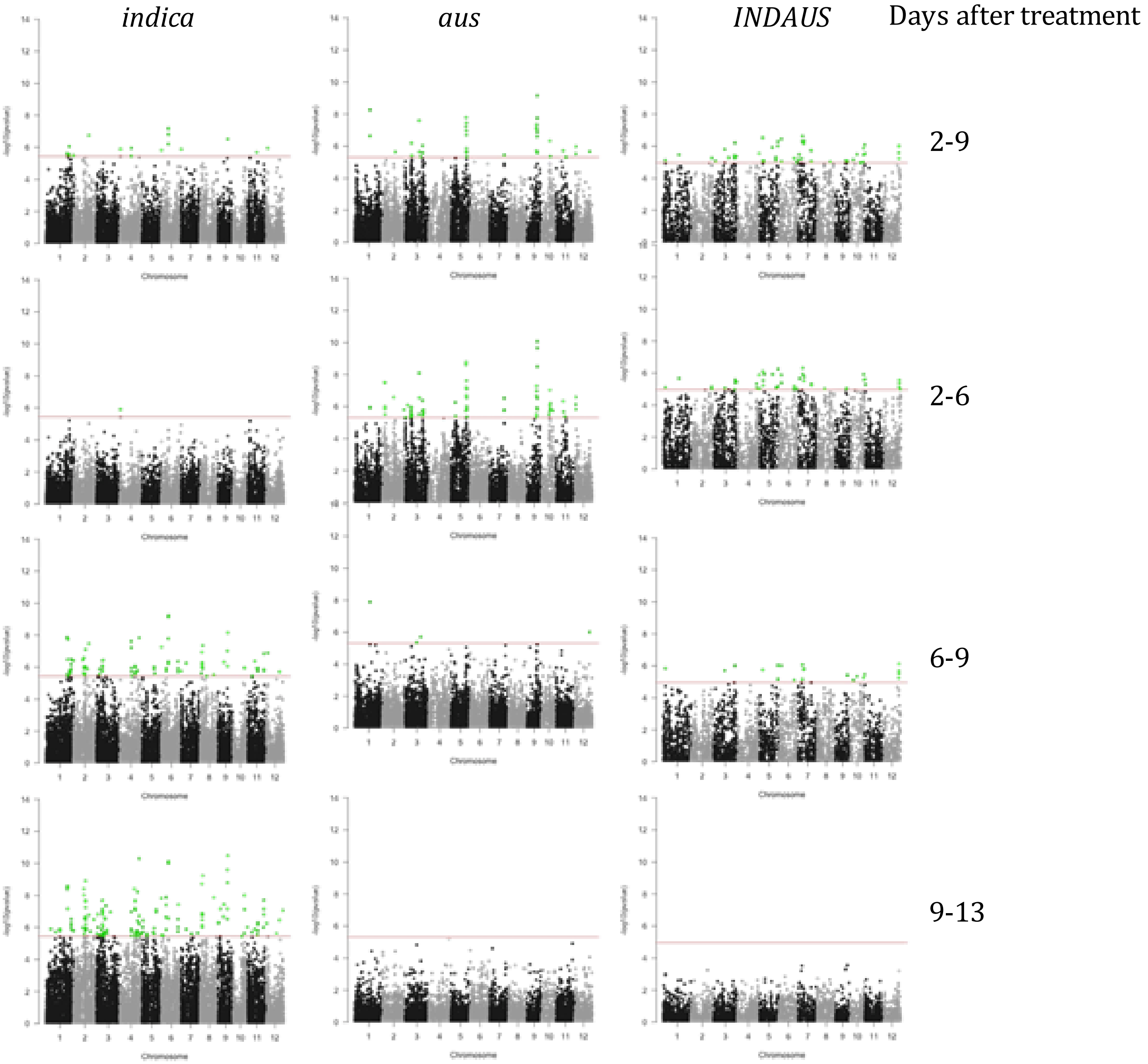

Model 'Marker-by-Treatment' for transpiration rate (TR)

e

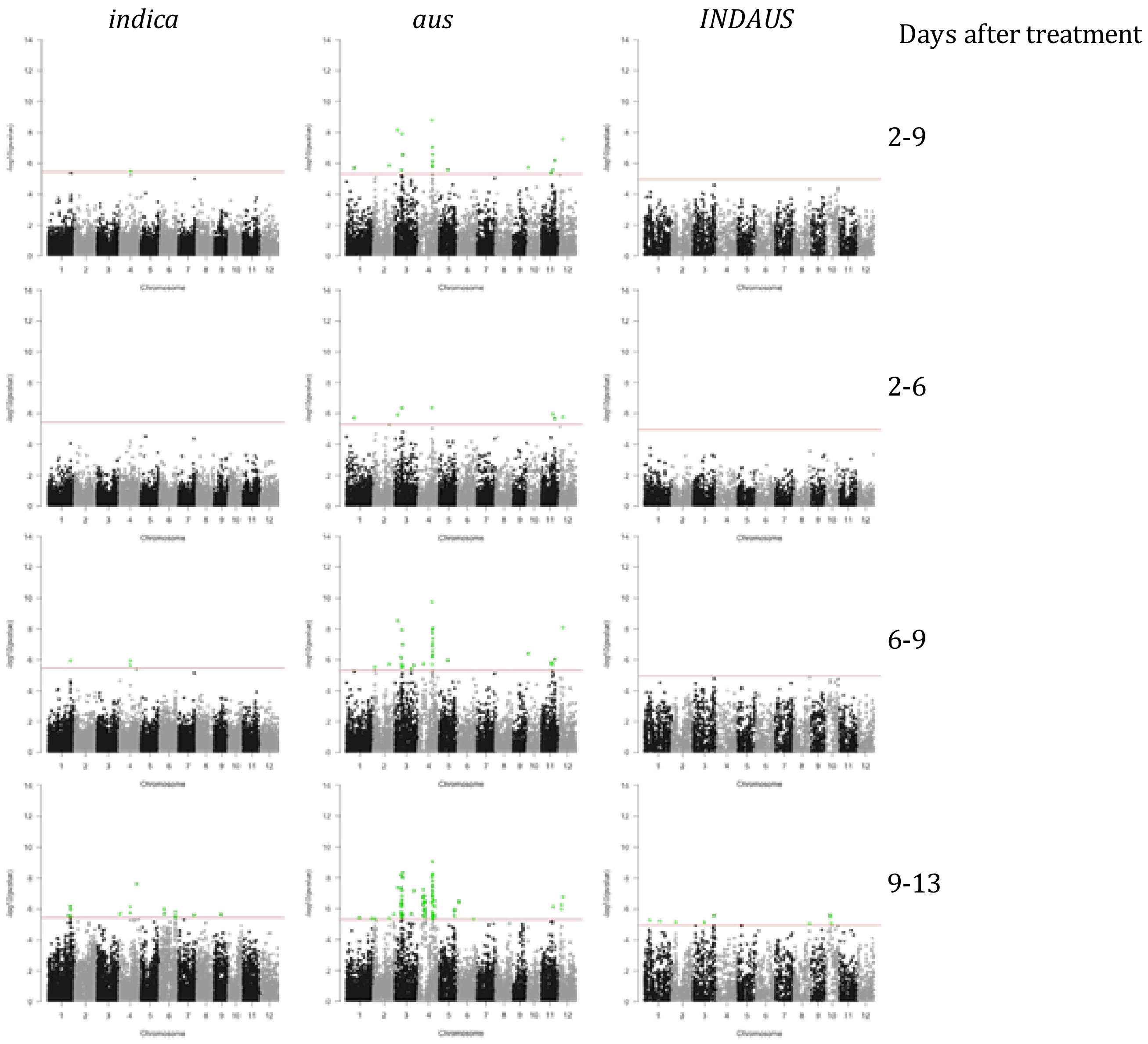

f Model ‘Marker-by-Treatment’ for transpiration use efficiency (TUE)

indica *aus* *INDAUS* Days after treatment

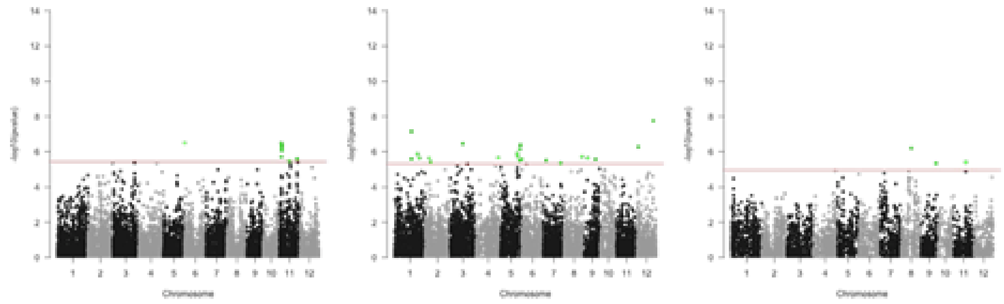

2-9

Remaining panels for other time intervals are included in the main body of the manuscript.

g

Mixed Linear Model for relative growth rate for stress tolerance index (RGR-STI)

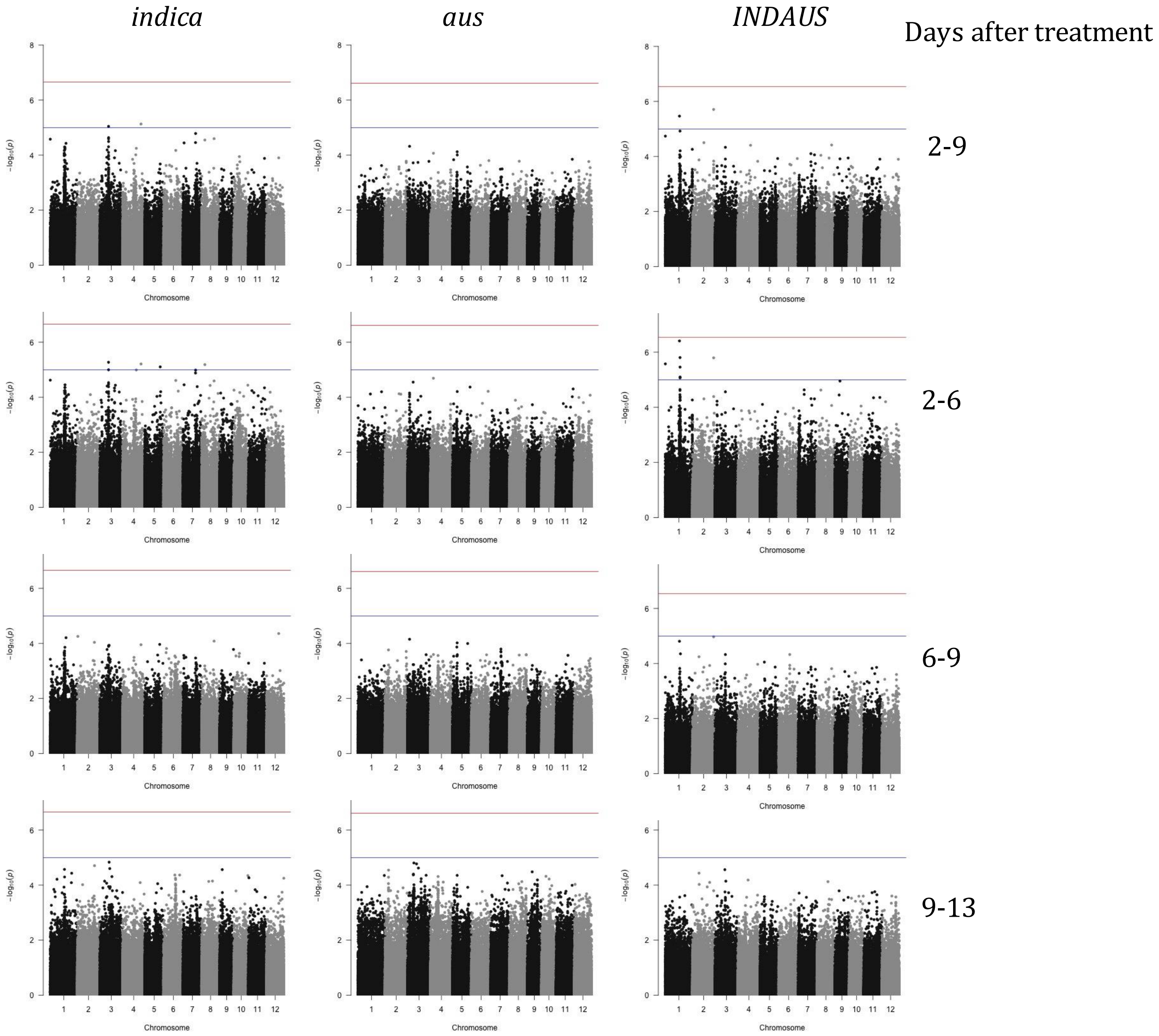

h Mixed Linear Model for transpiration rate of stress weighted performance index (TR-SWP)

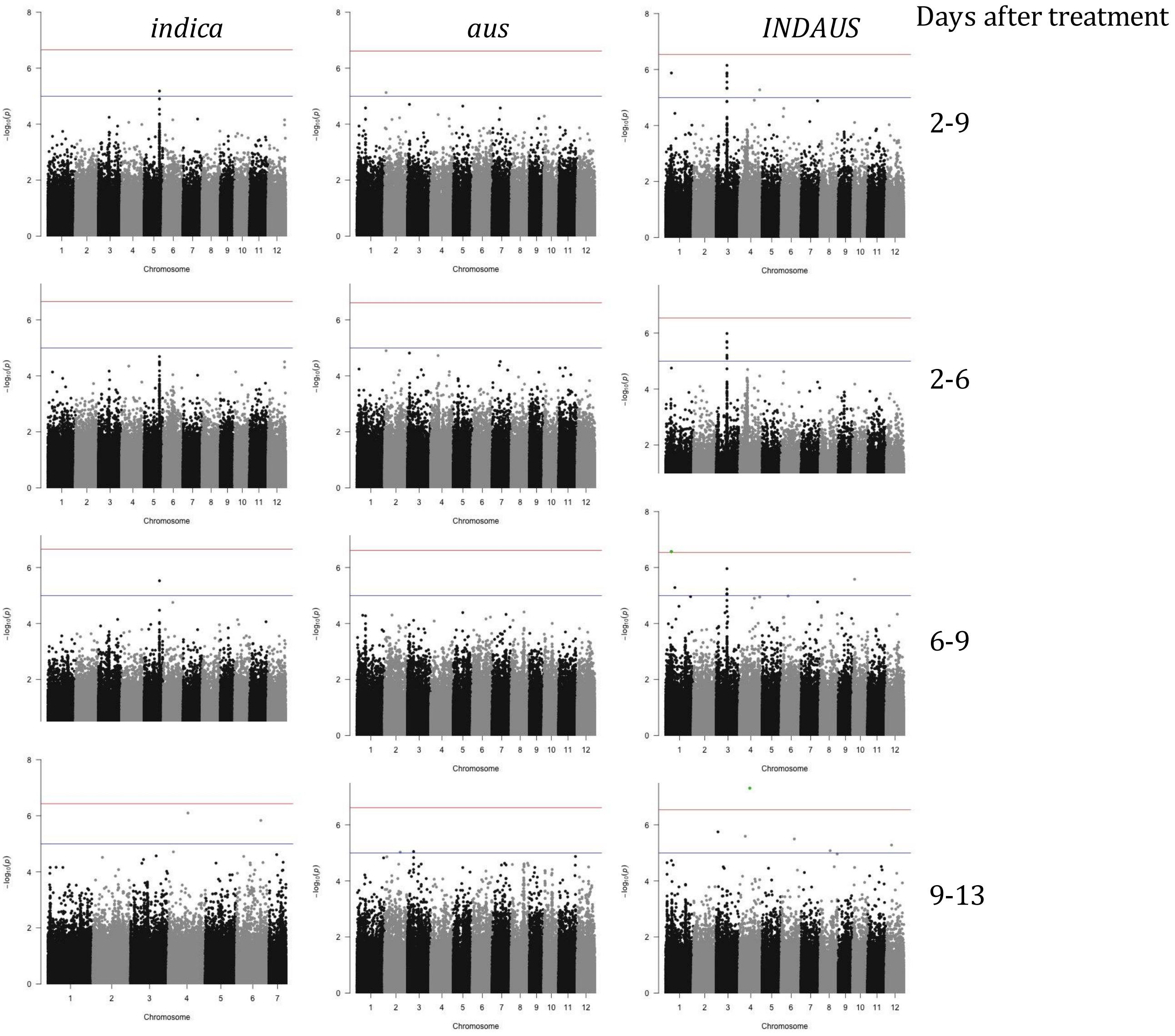

i Mixed Linear Model for transpiration use efficiency of TOL index (TUE-TOL)

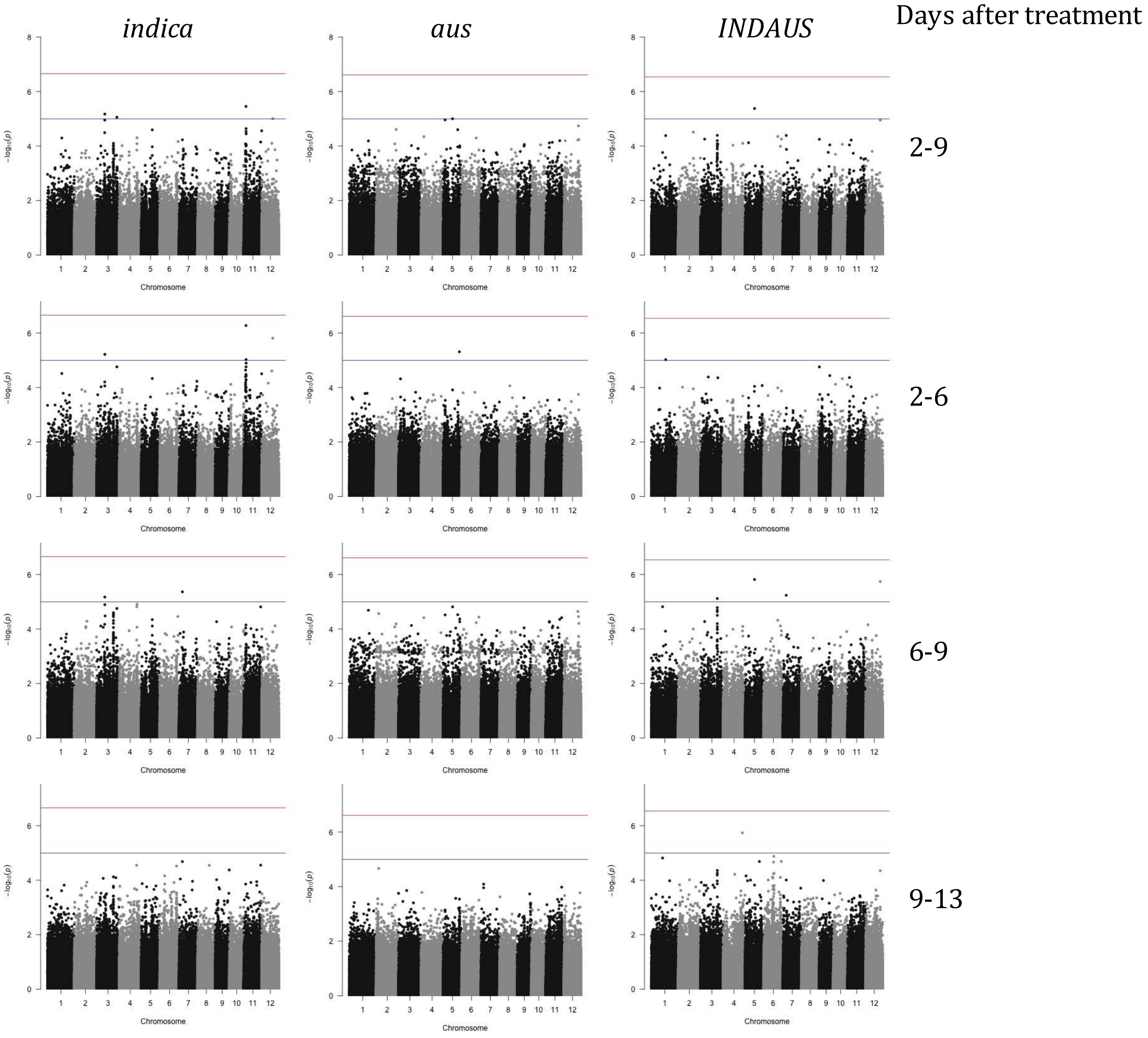

Supplementary Figure 5: Manhattan plots of SNPs for association with the traits relative growth rate, transpiration rate and transpiration use efficiency using both models (conventional Mixed Linear Model (MLM) and our developed Interaction model) at the four different intervals.

SNPs are highlighted in green if they reach genome wide significance for association with the trait at each time interval in *indica*, *aus* and *INDAUS* (panels left to right). SNPs associated with the trait are shown at the different time intervals: 2-9, 2-6, 6-9 and 9-13 days after treatment (panels top to bottom). (**a-c**) Manhattan plots using the GWAS interaction model 'Marker'. Horizontal red lines indicate Bonferroni significance thresholds $P < 2.6\times10^{-6}$, 3.0×10^{-6} and 9.0×10^{-6} for *indica*, *aus* and *INDAUS*, respectively. (**a**) SNPs associated with RGR, (**b**) TR and (**c**) TUE. (**d-f**) as in (**a-c**) with GWAS interaction model 'Marker-by-Treatment' and panels for TUE only for interval 2-9 DAST. (**g-i**) Manhattan plots using the GWAS MLM model in the software TASSEL. Horizontal red lines indicate Bonferroni significance thresholds $P < 2.2\times10^{-7}$, $\times10^{-6}$ and 2.9×10^{-7} for *indica*, *aus* and *INDAUS*, respectively. While blue lines indicate the suggestive threshold $P < 1\times10^{-5}$ (g) SNPs associated with relative growth rate of stress tolerance index (RGR-STI) index (**h**) with transpiration rate of stress-weighted performance (TR-SWP) (**i**) with transpiration use efficiency of tolerance index (TUE-TOL).

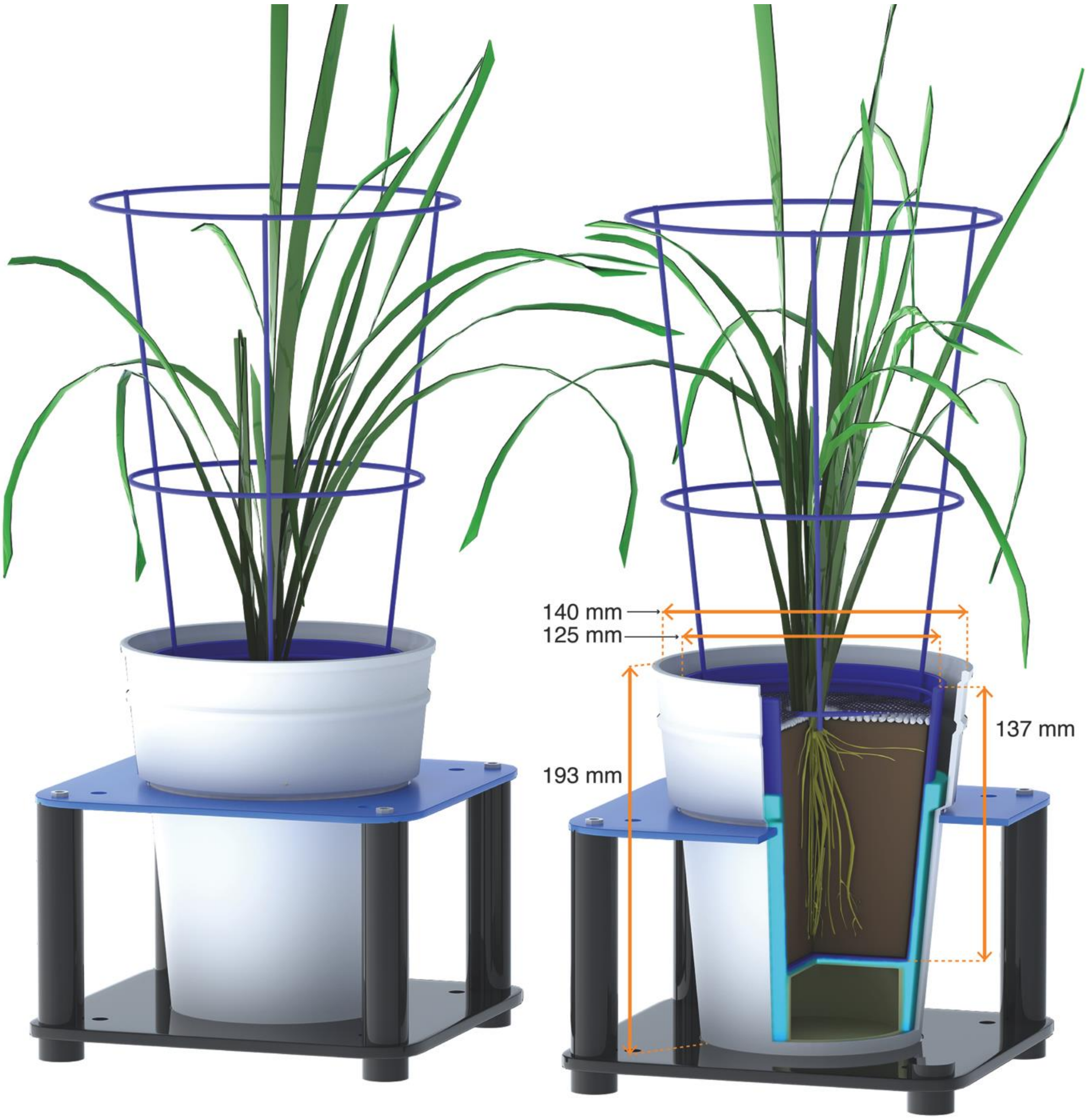

Supplementary Figure 6: **Double-pot design to enable growth of rice in controlled waterlogged conditions, to which salt could be applied.**

Dark blue pots (125 mm diameter, 137 mm height) with drainage holes were placed inside white closed base pots (140 mm diameter, 193 mm height). White pots had a sealed plastic container (93 mm diameter, 50 mm height) in the bottom to reduce the volume of water (light blue) required to flood the dark blue pot inside the white pot. The double-pot system was filled to a certain water level to create a waterlogged environment typical for rice growing conditions, while avoiding water spillage on the conveyor system. The soil surface was covered by 200 g of white gravel (approximately 2-5 mm particle size) to reduce development of algae and evaporation from the soil surface. Blue PVC mats were placed on top of the gravel to further

reduce evaporation. A blue frame was placed on top of pots in a fixed position to support the plant for easier image analysis.

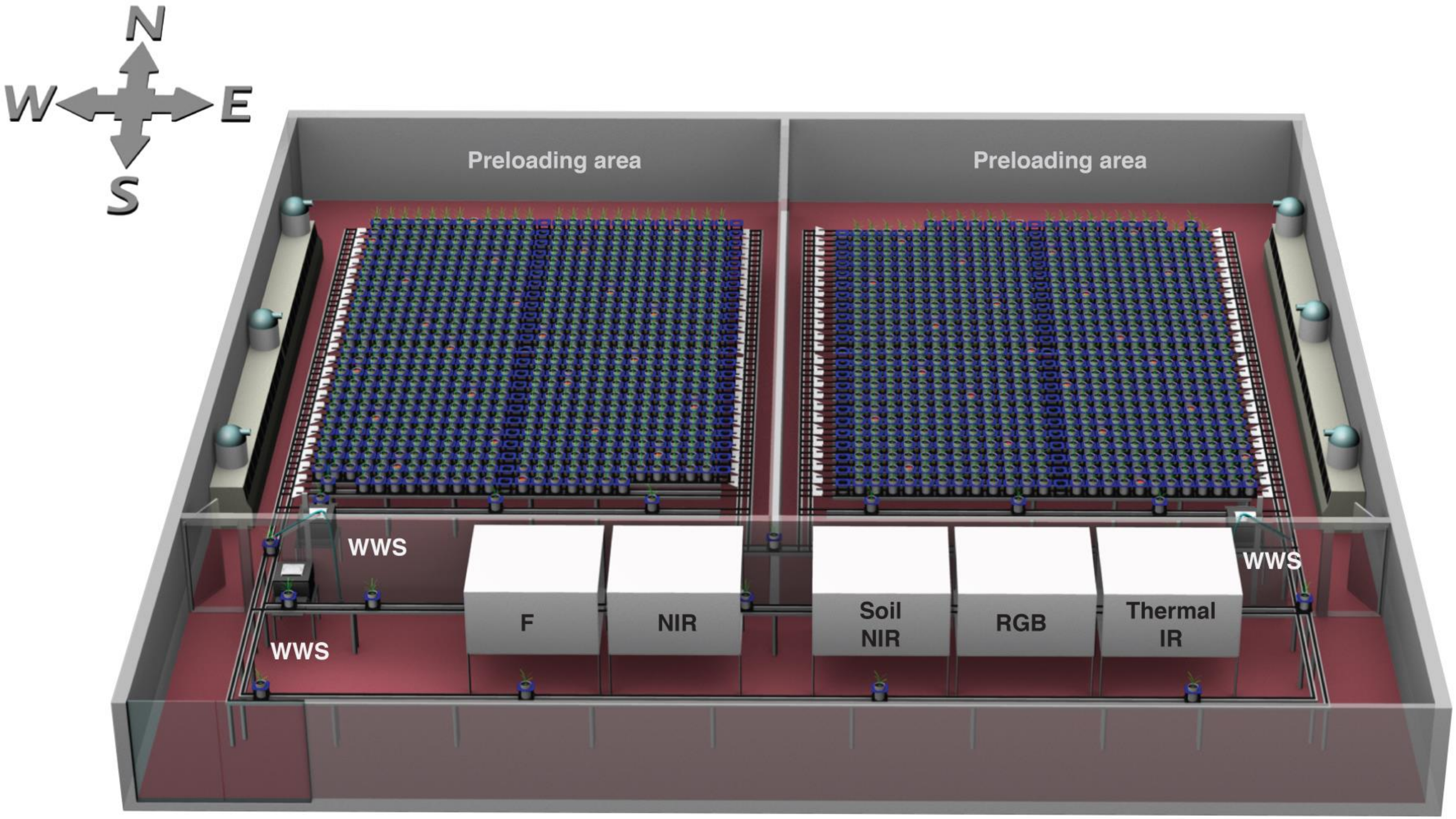

N
W
E
S
Preloading area
Preloading area
WWS
WWS
WWS
F
NIR
Soil NIR
RGB
Thermal IR

Supplementary Figure 7: Illustration of the two quarantine-approved Smarthouses at The Plant Accelerator® (Australian Plant Phenomics Facility, University of Adelaide, Adelaide, Australia; -34.97113, 138.63989).

Two Smarthouses, north-east (NE) and north-west (NW), fitted with conveyor systems are connected to one imaging hall with five camera stations (LemnaTec Scanalyzer 3D) for the daily non-destructive phenotyping of plants. The imaging stations are arranged in a 2 + 3 format from west to east, providing fluorescence imaging, near-infrared imaging, near-infrared imaging of pots (soil), red-green-blue visible imaging and far infrared (thermal) imaging. Each Smarthouse has a capacity of up to 624 carts. Two columns remained unused due to weight restrictions on the conveyor system. The carts marked in red (12 out of the 528 carts) represent the evaporation pots (pots without a plant). Air conditioners are on opposite sides of each Smarthouse and humidifiers are placed on top of the air conditioning units solely for this experiment. There are three watering and weighing stations (WWS). Each plant/carrier is delivered automatically on the conveyor system to the WWS and is controlled by high capacity computing equipment. The preloading area is where the plants are grown on benches before they are manually loaded onto the conveyor system. It should also be noted that there is early morning shading on the eastern side of the NE Smarthouse from adjacent trees and hillside. This shading is likely to be a cause of some spatial variation.

a ***indica***

NE Smarthouse Positions

Lanes	1	2	3	4	5	6	7	8	9	10	11	12	13	14	15	16	17	18	19	20	21	22	23	24	25	26
1	Void	177	177	219	219	191	191	Evap	166	166	52	52	Void	273	273	100	100	1	1	251	251	35	35	278	278	Void
2	295	295	69	69	107	107	293	293	13	13	249	249	Void	41	41	247	247	81	81	Evap	16	16	142	142	Void	Void
3	Void	Evap	206	206	272	272	106	106	157	157	158	158	Void	65	65	104	104	66	66	241	241	11	11	211	211	Void
4	95	95	282	282	300	300	161	161	179	179	240	240	Void	253	253	Evap	196	196	194	194	265	265	204	204	Void	Void
5	Void	19	19	213	213	39	39	14	14	Evap	172	172	Void	115	115	243	243	21	21	28	28	98	98	44	44	Void
6	146	146	88	88	242	242	62	62	4	4	254	254	Void	87	87	131	131	130	130	234	234	61	61	Evap	Void	Void
7	Void	252	252	Evap	114	114	71	71	112	112	276	276	Void	3	3	245	245	24	24	53	53	136	136	50	50	Void
8	122	122	289	289	55	55	258	258	250	250	129	129	Void	76	76	111	111	Evap	6	6	173	173	58	58	Void	Void
9	Void	240	240	2	2	33	33	193	193	150	150	Evap	Void	117	117	22	22	269	269	89	89	67	67	242	242	Void
10	140	140	158	158	111	111	298	298	85	85	163	163	Void	287	287	295	295	271	271	215	215	Evap	209	209	Void	Void
11	Void	203	203	216	216	Evap	28	28	221	221	50	50	Void	127	127	201	201	212	212	31	31	4	4	293	293	Void
12	54	54	151	151	169	169	202	202	35	35	266	266	Void	Evap	230	230	94	94	283	283	37	37	99	99	Void	Void
13	Void	254	254	270	270	149	149	Evap	68	68	188	188	Void	57	57	206	206	281	281	123	123	284	284	275	275	Void
14	292	292	214	214	187	187	170	170	102	102	161	161	Void	171	171	92	92	125	125	Evap	172	172	132	132	Void	Void
15	Void	Evap	119	119	291	291	56	56	256	256	101	101	Void	252	252	38	38	194	194	40	40	176	176	120	120	Void
16	247	247	77	77	43	43	168	168	173	173	231	231	Void	233	233	Evap	220	220	10	10	267	267	34	34	Void	Void
17	Void	138	138	141	141	294	294	239	239	Evap	73	73	Void	103	103	91	91	9	9	285	285	156	156	152	152	Void
18	80	80	26	26	180	180	6	6	83	83	226	226	Void	246	246	280	280	237	237	131	131	118	118	Evap	Void	Void
19	Void	179	179	Evap	249	249	186	186	134	134	84	84	Void	165	165	113	113	45	45	133	133	189	189	86	86	Void
20	16	16	97	97	74	74	238	238	296	296	116	116	Void	264	264	130	130	Evap	115	115	255	255	196	196	Void	Void
21	Void	17	17	279	279	75	75	183	183	135	135	Evap	Void	109	109	205	205	143	143	277	277	122	122	272	272	Void
22	29	29	244	244	288	288	41	41	229	229	268	268	Void	5	5	181	181	105	105	8	8	Evap	258	258	Void	Void
23	Void	108	108	12	12	Evap	204	204	251	251	147	147	Void	20	20	262	262	148	148	289	289	259	259	48	48	Void
24	96	96	25	25	182	182	299	299	55	55	166	166	Void	Evap	36	36	274	274	290	290	30	30	121	121	Void	Void

b ***indica***

NE Smarthouse Positions

Lanes	1	2	3	4	5	6	7	8	9	10	11	12	13	14	15	16	17	18	19	20	21	22	23	24	25	26
1	Void	1	2	1	2	2	1	Evap	2	1	2	1	Void	2	1	1	2	1	2	2	1	1	2	2	1	Void
2	2	1	2	1	1	2	1	2	1	2	1	2	Void	1	2	2	1	2	1	Evap	2	1	1	2	Void	Void
3	Void	Evap	1	2	1	2	1	2	1	2	2	1	Void	1	2	2	1	1	2	2	1	1	2	2	1	Void
4	2	1	1	2	1	2	2	1	1	2	2	1	Void	2	1	Evap	1	2	1	2	2	1	1	2	Void	Void
5	Void	1	2	1	2	2	1	2	1	Evap	1	2	Void	1	2	2	1	2	1	2	1	2	1	2	1	Void
6	2	1	2	1	1	2	2	1	1	2	2	1	Void	2	1	2	1	2	1	2	1	2	1	Evap	Void	Void
7	Void	2	1	Evap	1	2	1	2	1	2	2	1	Void	1	2	1	2	1	2	2	1	1	2	1	2	Void
8	2	1	1	2	1	2	2	1	2	1	2	1	Void	2	1	1	2	Evap	1	2	1	2	2	1	Void	Void
9	Void	2	1	1	2	2	1	2	1	1	2	Evap	Void	2	1	2	1	1	2	2	1	1	2	1	2	Void
10	2	1	2	1	2	1	2	1	1	2	2	1	Void	2	1	1	2	2	1	1	2	Evap	1	2	Void	Void
11	Void	2	1	1	2	Evap	2	1	2	1	1	2	Void	2	1	2	1	1	2	1	2	2	1	2	1	Void
12	2	1	2	1	1	2	1	2	1	2	2	1	Void	Evap	1	2	2	1	1	2	1	2	1	2	Void	Void
13	Void	1	2	2	1	2	1	Evap	2	1	1	2	Void	2	1	2	1	1	2	1	2	1	2	2	1	Void
14	1	2	1	2	1	2	1	2	1	2	2	1	Void	2	1	2	1	2	1	Evap	2	1	2	1	Void	Void
15	Void	Evap	1	2	2	1	2	1	1	2	1	2	Void	1	2	1	2	2	1	1	2	2	1	1	2	Void
16	1	2	1	2	2	1	1	2	2	1	2	1	Void	1	2	Evap	2	1	2	1	1	2	1	2	Void	Void
17	Void	2	1	2	1	1	2	1	2	Evap	1	2	Void	2	1	1	2	1	2	1	2	1	2	1	2	Void
18	2	1	1	2	1	2	2	1	1	2	2	1	Void	1	2	1	2	1	2	2	1	1	2	Evap	Void	Void
19	Void	1	2	Evap	2	1	2	1	2	1	2	1	Void	1	2	1	2	2	1	2	1	1	2	1	2	Void
20	1	2	1	2	1	2	1	2	2	1	2	1	Void	2	1	1	2	Evap	2	1	2	1	1	2	Void	Void
21	Void	2	1	2	1	1	2	1	2	1	2	Evap	Void	2	1	1	2	1	2	2	1	1	2	1	2	Void
22	2	1	1	2	1	2	1	2	1	2	2	1	Void	2	1	2	1	1	2	2	1	Evap	1	2	Void	Void
23	Void	2	1	1	2	Evap	1	2	1	2	1	2	Void	1	2	2	1	1	2	1	2	1	2	2	1	Void
24	1	2	2	1	1	2	2	1	2	1	1	2	Void	Evap	2	1	1	2	1	2	2	1	1	2	Void	Void

c

indica

NW Smarthouse Positions

Lanes	26	25	24	23	22	21	20	19	18	17	16	15	14	13	12	11	10	9	8	7	6	5	4	3	2	1
1	Void	280	280	243	243	200	200	190	190	203	203	178	178	Void	277	277	156	156	Evap	72	72	46	46	48	48	Void
2	Void	Void	183	183	2	2	Evap	187	187	241	241	59	59	Void	228	228	234	234	18	18	276	276	181	181	261	261
3	Void	26	26	104	104	177	177	120	120	68	68	271	271	Void	124	124	267	267	91	91	21	21	89	89	Evap	Void
4	Void	Void	71	71	51	51	69	69	214	214	Evap	207	207	Void	49	49	236	236	208	208	66	66	246	246	144	144
5	Void	232	232	222	222	163	163	134	134	284	284	170	170	Void	216	216	Evap	52	52	167	167	38	38	223	223	Void
6	Void	Void	Evap	146	146	3	3	102	102	58	58	182	182	Void	81	81	106	106	154	154	70	70	15	15	37	37
7	Void	5	5	159	159	110	110	239	239	279	279	39	39	Void	257	257	94	94	275	275	235	235	Evap	136	136	Void
8	Void	Void	79	79	148	148	97	97	Evap	105	105	218	218	Void	180	180	227	227	64	64	67	67	86	86	11	11
9	Void	160	160	225	225	62	62	213	213	142	142	248	248	Void	Evap	40	40	152	152	198	198	250	250	1	1	Void
10	Void	Void	168	168	Evap	85	85	75	75	164	164	121	121	Void	176	176	195	195	260	260	268	268	264	264	139	139
11	Void	25	25	286	286	22	22	23	23	145	145	31	31	Void	98	98	90	90	211	211	Evap	9	9	101	101	Void
12	Void	Void	273	273	300	300	230	230	42	42	53	53	Evap	Void	210	210	118	118	263	263	73	73	93	93	233	233
13	Void	60	60	294	294	192	192	162	162	298	298	288	288	Void	132	132	265	265	Evap	237	237	224	224	245	245	Void
14	Void	Void	270	270	226	226	Evap	24	24	278	278	14	14	Void	30	30	95	95	113	113	185	185	127	127	135	135
15	Void	171	171	47	47	229	229	27	27	63	63	61	61	Void	43	43	7	7	78	78	82	82	137	137	Evap	Void
16	Void	Void	117	117	221	221	175	175	17	17	Evap	74	74	Void	217	217	153	153	215	215	92	92	184	184	100	100
17	Void	223	223	82	82	269	269	70	70	189	189	235	235	Void	200	200	Evap	155	155	217	217	195	195	207	207	Void
18	Void	Void	Evap	165	165	256	256	149	149	151	151	285	285	Void	108	108	126	126	79	79	205	205	47	47	23	23
19	Void	208	208	12	12	140	140	18	18	291	291	78	78	Void	199	199	190	190	201	201	290	290	Evap	210	210	Void
20	Void	Void	119	119	93	93	84	84	Evap	225	225	125	125	Void	64	64	299	299	63	63	174	174	283	283	116	116
21	Void	54	54	143	143	7	7	33	33	160	160	46	46	Void	Evap	297	297	20	20	255	255	287	287	133	133	Void
22	Void	Void	227	227	Evap	198	198	144	144	32	32	10	10	Void	260	260	60	60	123	123	145	145	178	178	159	159
23	Void	261	261	45	45	141	141	34	34	128	128	192	192	Void	202	202	236	236	147	147	Evap	222	222	175	175	Void
24	Void	Void	153	153	36	36	197	197	259	259	99	99	Evap	Void	286	286	244	244	83	83	220	220	57	57	232	232

d

indica

NW Smarthouse Positions

Lanes	26	25	24	23	22	21	20	19	18	17	16	15	14	13	12	11	10	9	8	7	6	5	4	3	2	1
1	Void	1	2	2	1	1	2	2	1	1	2	1	2	Void	2	1	1	2	Evap	2	1	1	2	2	1	Void
2	Void	Void	1	2	2	1	Evap	1	2	1	2	2	1	Void	1	2	2	1	1	2	1	2	1	2	1	2
3	Void	2	1	2	1	2	1	2	1	2	1	1	2	Void	1	2	1	2	2	1	2	1	1	2	Evap	Void
4	Void	Void	1	2	1	2	1	2	1	2	Evap	1	2	Void	2	1	1	2	1	2	2	1	2	1	1	2
5	Void	1	2	1	2	2	1	2	1	2	1	2	1	Void	2	1	Evap	2	1	1	2	2	1	1	2	Void
6	Void	Void	Evap	2	1	2	1	1	2	1	2	2	1	Void	1	2	1	2	1	2	2	1	2	1	1	2
7	Void	2	1	1	2	1	2	1	2	1	2	1	2	Void	2	1	1	2	2	1	1	2	Evap	2	1	Void
8	Void	Void	2	1	2	1	1	2	Evap	1	2	2	1	Void	1	2	2	1	1	2	2	1	2	1	2	1
9	Void	1	2	2	1	1	2	2	1	2	1	1	2	Void	Evap	1	2	1	2	2	1	1	2	1	2	Void
10	Void	Void	1	2	Evap	2	1	2	1	2	1	1	2	Void	1	2	2	1	1	2	1	2	1	2	2	1
11	Void	2	1	1	2	2	1	2	1	2	1	1	2	Void	2	1	2	1	1	2	Evap	2	1	1	2	Void
12	Void	Void	2	1	2	1	2	1	2	1	1	2	Evap	Void	2	1	1	2	1	2	2	1	1	2	2	1
13	Void	2	1	2	1	1	2	1	2	2	1	2	1	Void	1	2	1	2	Evap	1	2	1	2	2	1	Void
14	Void	Void	1	2	1	2	Evap	1	2	1	2	1	2	Void	1	2	1	2	1	2	2	1	1	2	1	2
15	Void	2	1	2	1	1	2	2	1	1	2	2	1	Void	1	2	1	2	2	1	1	2	1	2	Evap	Void
16	Void	Void	2	1	1	2	1	2	1	2	Evap	2	1	Void	2	1	2	1	1	2	1	2	2	1	2	1
17	Void	1	2	1	2	1	2	2	1	2	1	1	2	Void	1	2	Evap	1	2	2	1	1	2	2	1	Void
18	Void	Void	Evap	2	1	2	1	2	1	2	1	1	2	Void	2	1	2	1	2	1	1	2	1	2	2	1
19	Void	2	1	2	1	2	1	1	2	2	1	2	1	Void	2	1	2	1	1	2	1	2	Evap	2	1	Void
20	Void	Void	2	1	1	2	1	2	Evap	1	2	2	1	Void	2	1	2	1	1	2	1	2	2	1	2	1
21	Void	2	1	2	1	2	1	1	2	2	1	2	1	Void	Evap	1	2	1	2	1	2	2	1	2	1	Void
22	Void	Void	1	2	Evap	2	1	2	1	2	1	2	1	Void	1	2	2	1	2	1	2	1	1	2	1	2
23	Void	2	1	1	2	1	2	1	2	2	1	2	1	Void	1	2	2	1	1	2	Evap	2	1	2	1	Void
24	Void	Void	1	2	1	2	1	2	2	1	1	2	Evap	Void	2	1	2	1	2	1	2	1	1	2	1	2

e

aus

NE Smarthouse Positions

Lanes	1	2	3	4	5	6	7	8	9	10	11	12	13	14	15	16	17	18	19	20	21	22	23	24	25	26
1	Void	226	226	225	225	65	65	Evap	111	111	237	237	Void	195	195	68	68	89	89	73	73	95	95	198	198	Void
2	16	16	61	61	30	30	115	115	86	86	145	145	Void	176	176	161	161	110	110	Evap	163	163	33	33	Void	Void
3	Void	Evap	3	3	57	57	197	197	200	200	217	217	Void	102	102	103	103	44	44	126	126	251	251	6	6	Void
4	75	75	99	99	159	159	208	208	141	141	133	133	Void	90	90	Evap	210	210	40	40	240	240	194	194	Void	Void
5	Void	178	178	20	20	120	120	83	83	Evap	96	96	Void	59	59	100	100	157	157	245	245	81	81	84	84	Void
6	193	193	125	125	15	15	104	104	38	38	228	228	Void	52	52	144	144	180	180	9	9	5	5	Evap	Void	Void
7	Void	196	196	Evap	255	255	106	106	147	147	224	224	Void	1	1	24	24	36	36	98	98	136	136	62	62	Void
8	257	257	17	17	177	177	54	54	181	181	148	148	Void	29	29	137	137	Evap	101	101	223	223	27	27	Void	Void
9	Void	233	233	118	118	240	240	185	185	87	87	Evap	Void	65	65	254	254	45	45	137	137	14	14	172	172	Void
10	191	191	203	203	72	72	259	259	230	230	165	165	Void	236	236	200	200	123	123	149	149	Evap	238	238	Void	Void
11	Void	198	198	244	244	Evap	70	70	31	31	151	151	Void	121	121	164	164	171	171	106	106	250	250	217	217	Void
12	134	134	162	162	105	105	181	181	152	152	150	150	Void	Evap	28	28	194	194	112	112	10	10	108	108	Void	Void
13	Void	232	232	129	129	78	78	Evap	21	21	192	192	Void	110	110	246	246	91	91	55	55	227	227	32	32	Void
14	145	145	8	8	229	229	220	220	221	221	199	199	Void	153	153	216	216	215	215	Evap	197	197	214	214	Void	Void
15	Void	Evap	50	50	176	176	12	12	179	179	180	180	Void	206	206	18	18	225	225	42	42	224	224	96	96	Void
16	160	160	74	74	27	27	1	1	205	205	9	9	Void	16	16	Evap	83	83	80	80	22	22	88	88	Void	Void
17	Void	239	239	127	127	109	109	13	13	Evap	7	7	Void	218	218	81	81	19	19	202	202	256	256	23	23	Void
18	24	24	77	77	64	64	5	5	34	34	11	11	Void	174	174	163	163	184	184	166	166	226	226	Evap	Void	Void
19	Void	33	33	Evap	132	132	131	131	201	201	196	196	Void	187	187	177	177	154	154	146	146	2	2	252	252	Void
20	53	53	79	79	183	183	222	222	82	82	219	219	Void	204	204	211	211	Evap	116	116	20	20	186	186	Void	Void
21	Void	103	103	4	4	29	29	76	76	61	61	Evap	Void	43	43	107	107	175	175	111	111	133	133	36	36	Void
22	59	59	168	168	182	182	247	247	257	257	136	136	Void	25	25	122	122	44	44	212	212	Evap	115	115	Void	Void
23	Void	135	135	113	113	Evap	253	253	93	93	167	167	Void	37	37	139	139	69	69	155	155	158	158	63	63	Void
24	231	231	38	38	207	207	71	71	241	241	119	119	Void	Evap	41	41	49	49	243	243	242	242	39	39	Void	Void

f

aus

NE Smarthouse Positions

Lanes	1	2	3	4	5	6	7	8	9	10	11	12	13	14	15	16	17	18	19	20	21	22	23	24	25	26
1	Void	2	1	1	2	1	2	Evap	2	1	2	1	Void	2	1	1	2	1	2	2	1	1	2	1	2	Void
2	2	1	2	1	1	2	2	1	2	1	1	2	Void	2	1	2	1	2	1	Evap	2	1	2	1	Void	Void
3	Void	Evap	1	2	1	2	1	2	2	1	2	1	Void	1	2	2	1	1	2	2	1	1	2	1	2	Void
4	1	2	2	1	1	2	2	1	1	2	2	1	Void	2	1	Evap	2	1	2	1	1	2	2	1	Void	Void
5	Void	2	1	2	1	2	1	2	1	Evap	1	2	Void	1	2	1	2	2	1	2	1	1	2	1	2	Void
6	2	1	2	1	1	2	1	2	1	2	2	1	Void	2	1	2	1	1	2	1	2	2	1	Evap	Void	Void
7	Void	1	2	Evap	2	1	2	1	1	2	1	2	Void	2	1	1	2	2	1	2	1	1	2	2	1	Void
8	1	2	1	2	2	1	1	2	1	2	2	1	Void	2	1	1	2	Evap	1	2	1	2	1	2	Void	Void
9	Void	1	2	1	2	2	1	2	1	2	1	Evap	Void	1	2	2	1	1	2	1	2	2	1	1	2	Void
10	2	1	1	2	1	2	2	1	1	2	2	1	Void	2	1	1	2	1	2	2	1	Evap	1	2	Void	Void
11	Void	1	2	1	2	Evap	2	1	1	2	2	1	Void	1	2	2	1	2	1	2	1	2	1	1	2	Void
12	2	1	2	1	1	2	2	1	2	1	1	2	Void	Evap	1	2	2	1	1	2	1	2	1	2	Void	Void
13	Void	2	1	1	2	1	2	Evap	2	1	2	1	Void	2	1	1	2	1	2	2	1	2	1	1	2	Void
14	2	1	2	1	2	1	1	2	2	1	1	2	Void	1	2	1	2	1	2	Evap	2	1	1	2	Void	Void
15	Void	Evap	1	2	1	2	1	2	2	1	1	2	Void	1	2	2	1	2	1	1	2	1	2	2	1	Void
16	1	2	1	2	2	1	1	2	1	2	1	2	Void	2	1	Evap	1	2	2	1	2	1	1	2	Void	Void
17	Void	2	1	1	2	2	1	1	2	Evap	2	1	Void	2	1	1	2	1	2	1	2	2	1	2	1	Void
18	1	2	1	2	2	1	1	2	1	2	2	1	Void	1	2	1	2	1	2	2	1	1	2	Evap	Void	Void
19	Void	2	1	Evap	2	1	2	1	1	2	2	1	Void	2	1	2	1	2	1	1	2	1	2	2	1	Void
20	1	2	2	1	2	1	1	2	2	1	1	2	Void	2	1	2	1	Evap	1	2	2	1	2	1	Void	Void
21	Void	1	2	2	1	2	1	1	2	2	1	Evap	Void	1	2	1	2	2	1	1	2	1	2	2	1	Void
22	1	2	1	2	1	2	2	1	2	1	2	1	Void	1	2	2	1	1	2	2	1	Evap	2	1	Void	Void
23	Void	2	1	1	2	Evap	2	1	1	2	2	1	Void	1	2	1	2	2	1	1	2	1	2	2	1	Void
24	1	2	1	2	1	2	2	1	2	1	1	2	Void	Evap	2	1	2	1	2	1	2	1	2	1	Void	Void

g

aus

NW Smarthouse Positions

Lanes	26	25	24	23	22	21	20	19	18	17	16	15	14	13	12	11	10	9	8	7	6	5	4	3	2	1
1	Void	79	79	231	231	30	30	209	209	14	14	147	147	Void	245	245	91	91	Evap	236	236	237	237	148	148	Void
2	Void	Void	167	167	51	51	Evap	105	105	125	125	243	243	Void	101	101	43	43	163	163	204	204	239	239	254	254
3	Void	47	47	189	189	66	66	134	134	62	62	155	155	Void	203	203	188	188	157	157	227	227	140	140	Evap	Void
4	Void	Void	170	170	89	89	247	247	162	162	Evap	259	259	Void	232	232	126	126	173	173	206	206	48	48	242	242
5	Void	141	141	31	31	78	78	2	2	178	178	123	123	Void	220	220	Evap	45	45	228	228	25	25	112	112	Void
6	Void	Void	Evap	219	219	255	255	248	248	21	21	143	143	Void	132	132	202	202	68	68	39	39	90	90	152	152
7	Void	246	246	113	113	75	75	72	72	95	95	144	144	Void	124	124	56	56	88	88	221	221	Evap	223	223	Void
8	Void	Void	52	52	46	46	201	201	Evap	214	214	60	60	Void	120	120	73	73	142	142	10	10	230	230	116	116
9	Void	165	165	82	82	174	174	12	12	169	169	23	23	Void	Evap	94	94	63	63	85	85	137	137	22	22	Void
10	Void	Void	258	258	Evap	181	181	49	49	67	67	74	74	Void	161	161	58	58	6	6	195	195	135	135	117	117
11	Void	234	234	168	168	76	76	50	50	146	146	57	57	Void	69	69	183	183	192	192	Evap	97	97	208	208	Void
12	Void	Void	8	8	35	35	156	156	106	106	53	53	Evap	Void	99	99	173	173	256	256	86	86	213	213	41	41
13	Void	3	3	18	18	211	211	131	131	138	138	244	244	Void	40	40	87	87	Evap	32	32	28	28	218	218	Void
14	Void	Void	17	17	154	154	Evap	92	92	114	114	55	55	Void	235	235	172	172	128	128	100	100	102	102	104	104
15	Void	139	139	199	199	190	190	4	4	193	193	71	71	Void	85	85	210	210	251	251	84	84	98	98	Evap	Void
16	Void	Void	54	54	130	130	122	122	26	26	Evap	159	159	Void	186	186	129	129	42	42	249	249	119	119	250	250
17	Void	209	209	128	128	109	109	48	48	121	121	108	108	Void	51	51	Evap	153	153	66	66	205	205	234	234	Void
18	Void	Void	Evap	58	58	15	15	127	127	130	130	47	47	Void	184	184	35	35	169	169	233	233	187	187	216	216
19	Void	13	13	97	97	11	11	39	39	215	215	188	188	Void	80	80	164	164	149	149	46	46	Evap	172	172	Void
20	Void	Void	25	25	207	207	229	229	Evap	160	160	179	179	Void	156	156	189	189	19	19	37	37	92	92	94	94
21	Void	77	77	107	107	117	117	158	158	170	170	142	142	Void	Evap	249	249	124	124	235	235	171	171	248	248	Void
22	Void	Void	206	206	Evap	143	143	56	56	140	140	151	151	Void	118	118	190	190	60	60	212	212	34	34	67	67
23	Void	213	213	138	138	114	114	191	191	150	150	93	93	Void	182	182	241	241	258	258	Evap	166	166	26	26	Void
24	Void	Void	175	175	232	232	74	74	222	222	253	253	Evap	Void	185	185	64	64	252	252	7	7	70	70	238	238

h

aus

NW Smarthouse Positions

Lanes	26	25	24	23	22	21	20	19	18	17	16	15	14	13	12	11	10	9	8	7	6	5	4	3	2	1
1	Void	2	1	1	2	1	2	1	2	1	2	1	2	Void	2	1	2	1	Evap	2	1	1	2	1	2	Void
2	Void	Void	2	1	1	2	Evap	2	1	1	2	2	1	Void	2	1	1	2	1	2	2	1	1	2	2	1
3	Void	1	2	2	1	2	1	2	1	2	1	2	1	Void	2	1	2	1	2	1	2	1	2	1	Evap	Void
4	Void	Void	1	2	1	2	2	1	1	2	Evap	2	1	Void	2	1	2	1	1	2	1	2	1	2	1	2
5	Void	1	2	2	1	1	2	2	1	1	2	2	1	Void	1	2	Evap	1	2	1	2	1	2	1	2	Void
6	Void	Void	Evap	2	1	2	1	1	2	1	2	1	2	Void	2	1	1	2	2	1	2	1	1	2	1	2
7	Void	1	2	1	2	1	2	1	2	1	2	1	2	Void	1	2	2	1	1	2	2	1	Evap	1	2	Void
8	Void	Void	2	1	1	2	2	1	Evap	2	1	1	2	Void	2	1	2	1	1	2	1	2	2	1	1	2
9	Void	2	1	2	1	1	2	2	1	2	1	2	1	Void	Evap	1	2	1	2	2	1	2	1	1	2	Void
10	Void	Void	2	1	Evap	2	1	2	1	1	2	1	2	Void	1	2	2	1	1	2	2	1	2	1	1	2
11	Void	2	1	2	1	2	1	1	2	1	2	2	1	Void	1	2	1	2	1	2	Evap	1	2	1	2	Void
12	Void	Void	1	2	2	1	1	2	1	2	1	2	Evap	Void	1	2	2	1	1	2	1	2	2	1	1	2
13	Void	2	1	2	1	2	1	2	1	2	1	1	2	Void	1	2	1	2	Evap	1	2	1	2	2	1	Void
14	Void	Void	1	2	1	2	Evap	1	2	1	2	2	1	Void	2	1	2	1	2	1	2	1	1	2	2	1
15	Void	2	1	2	1	2	1	2	1	2	1	1	2	Void	1	2	1	2	2	1	2	1	1	2	Evap	Void
16	Void	Void	2	1	2	1	1	2	1	2	Evap	1	2	Void	2	1	2	1	2	1	2	1	1	2	1	2
17	Void	1	2	1	2	2	1	2	1	2	1	2	1	Void	1	2	Evap	1	2	2	1	2	1	2	1	Void
18	Void	Void	Evap	2	1	2	1	2	1	1	2	1	2	Void	1	2	2	1	2	1	2	1	2	1	1	2
19	Void	1	2	1	2	2	1	2	1	2	1	2	1	Void	1	2	1	2	1	2	1	2	Evap	1	2	Void
20	Void	Void	1	2	1	2	1	2	Evap	2	1	2	1	Void	2	1	2	1	2	1	1	2	1	2	2	1
21	Void	2	1	1	2	1	2	1	2	1	2	2	1	Void	Evap	1	2	2	1	1	2	1	2	1	2	Void
22	Void	Void	2	1	Evap	2	1	1	2	2	1	2	1	Void	2	1	1	2	2	1	2	1	2	1	1	2
23	Void	2	1	1	2	1	2	2	1	2	1	1	2	Void	2	1	2	1	2	1	Evap	1	2	1	2	Void
24	Void	Void	1	2	2	1	1	2	1	2	1	2	Evap	Void	2	1	1	2	1	2	2	1	2	1	1	2

Supplementary Figure 8: Conveyor layout of plants for both the *indica* and *aus* panels.

(**a**) The conveyor layout for the *indica* panel in the NE Smarthouse. The numbers in this figure represent the line numbers from 1 to 300. The replicated lines are colored

grey and the unreplicated lines are blue. Void carts are empty carts and Evap carts are evaporation carts. (**b**) The treatment layout for the *indica* panel in the NE Smarthouse. 1, control (no salt); 2, added salt; Void, empty carts; Evap, evaporation carts. (**c**) As **a**, with *indica* panel in the NW Smarthouse. (**d**) As **b**, with *indica* panel in the NW Smarthouse. (**e**) The conveyor layout for the *aus* panel in the NE Smarthouse. The numbers in this figure represent the line numbers from 1 to 259. The duplicated lines are colored grey and the thrice-replicated lines are blue. (**f**) As **d**, with treatment layout for the *aus* panel in the NE Smarthouse. (**g**) As **e**, with the conveyor layout for the *aus* panel in the NW Smarthouse. (**h**) As **f**, with treatment layout for the *aus* panel in the NW Smarthouse.

a

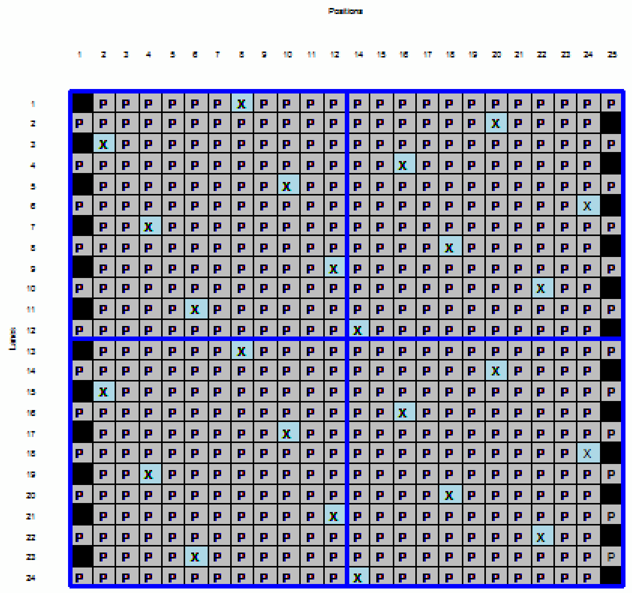

b

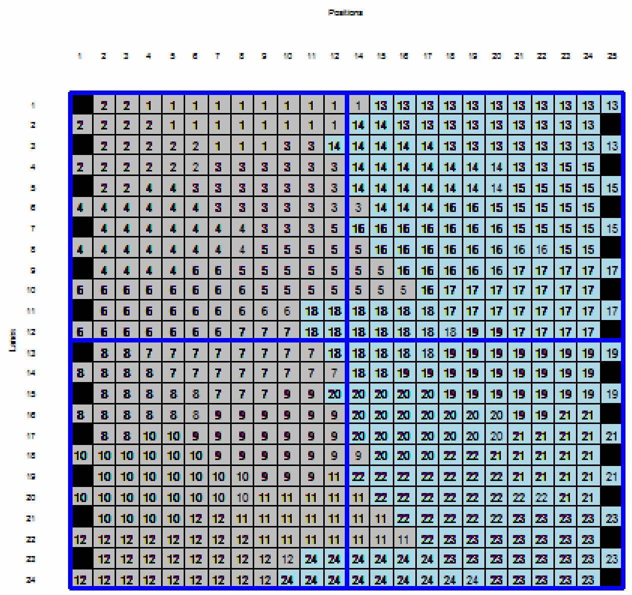

Supplementary Figure 9: Arrangement of evaporation carts

(a) One evaporation cart (blue X) amongst plant carts (P) was placed in every lane. The evaporation carts occurred between main plots, the odd lanes having a cart in positions 1–12 and the even lanes having a cart in positions 14–25. **(b)** Number of the closest evaporation cart for each cart. Evaporation carts are numbered going down the

left side and then down the right side. This information is used for spatial corrections of T and TUE.

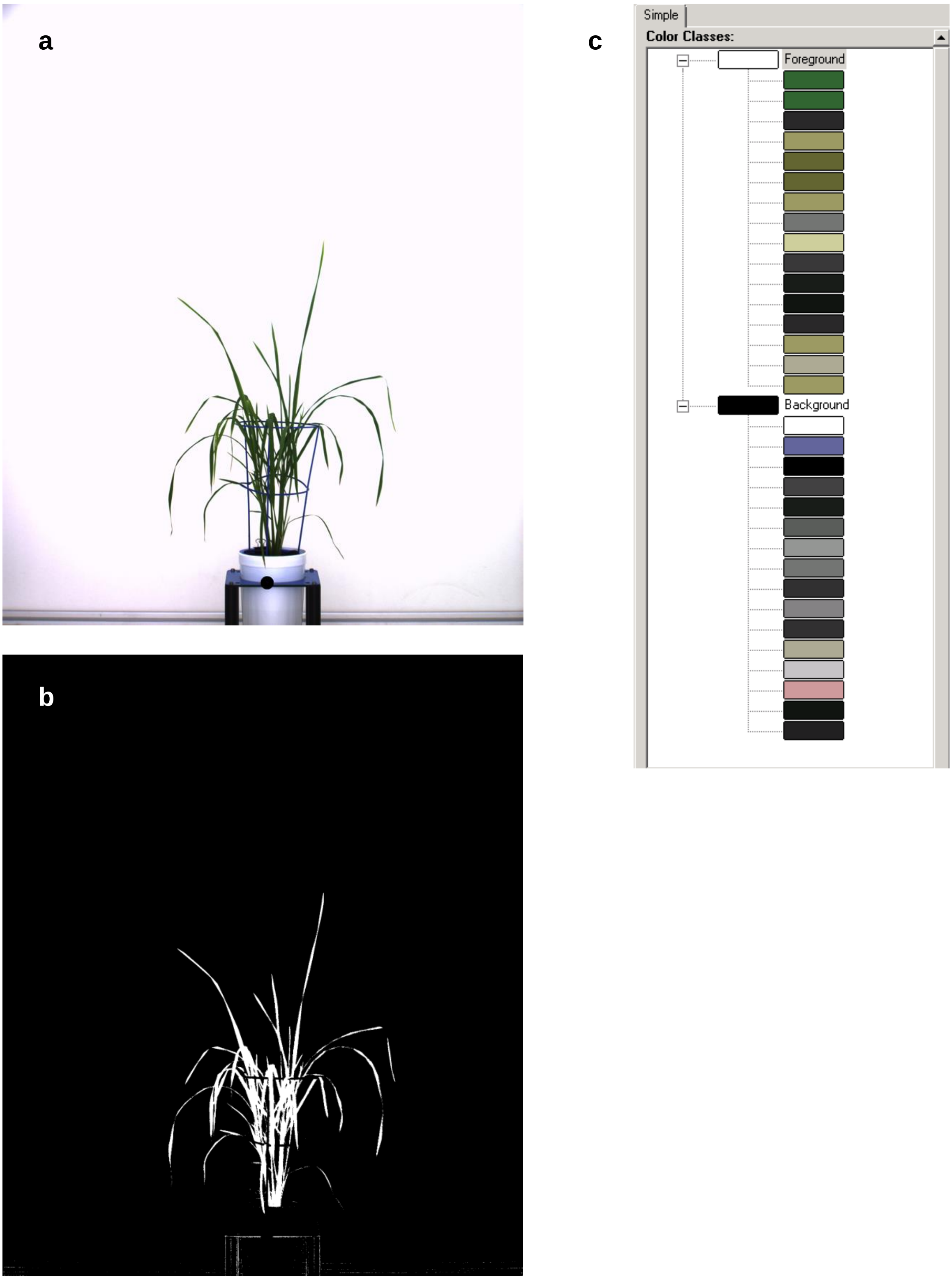

Supplementary Figure 10: Image analysis differentiates foreground (plant pixels) from the background (non-plant pixels).

(**a**) Example image of RGB side view. (**b**) Object extraction of the plant. (**c**) Color classes used to define foreground and background pixels from RGB images for the LemnaGrid Nearest Neighbor foreground-background color separation method.

Supplementary Table 1: Summary statistics of average percentage decrease by the salt treatment in both *indica* and *aus* for relative growth rate (RGR), transpiration rate (TR) and transpiration use efficiency (TUE). Minimum values, maximum values, standard errors (SE), standard deviation (SD) and 95% confidence interval (CI) are also displayed.

	indica						*aus*					
Trait/ interval DAS	Average % decrease	Min	Max	SE	SD	95% CI	Average % decrease	Min	Max	SE	SD	95% CI
**RGR.2to9**	**28.0**	-5.66	57.0	0.63	10.8	0.39	**36.1**	8.30	56.9	0.60	8.80	1.09
**RGR.2to6**	**20.9**	-10.59	48.6	0.57	9.8	0.36	**29.0**	2.50	51.9	0.60	9.00	1.11
**RGR.6to9**	**39.1**	0.84	75.3	0.78	13.3	0.48	**47.2**	9.10	74.3	0.60	10.1	1.24
**RGR.9to13**	**48.7**	4.86	86.1	0.87	14.9	0.54	**47.6**	11.6	83.2	0.70	11.9	1.46
**TR.2to9**	**20.8**	-37.23	58.4	0.73	12.4	0.45	**30.4**	-32.7	53.1	0.70	11.14	1.38
**TR.2to6**	**12.2**	-39.63	52.9	0.75	12.9	0.47	**15.8**	-51.3	44.1	0.77	12.21	1.51
**TR.6to9**	**29.4**	-38.75	63.8	0.73	12.5	0.45	**41.3**	-18.8	63.3	0.68	10.86	1.34
**TR.9to13**	**48.5**	-34.92	73.9	0.67	11.5	0.42	**57.4**	17.3	73.0	0.51	8.10	1.00
**TUE.2to9**	**23.3**	-3.45	57.9	0.68	11.7	0.42	**28.5**	3.12	58.0	0.62	9.90	1.22
**TUE.2to6**	**16.7**	-11.89	48.1	0.61	10.3	0.38	**24.4**	1.14	48.1	0.57	9.09	1.12
**TUE.6to9**	**30.8**	-2.09	73.1	0.85	14.6	0.53	**35.1**	-6.05	72.3	0.80	12.70	1.57
**TUE.9to13**	**33.3**	-12.52	82.7	1.13	19.3	0.70	**28.1**	-55.4	77.5	1.26	19.96	2.46

Supplementary Table 2: Percentage decrease of relative growth rate and early growth response index (EGRI) for frequently studied rice varieties ‘Pokkali’ and ‘IR28’, both in the *indica* subpopulation.

‘Pokkali’ is found in the literature to be a salt-tolerant variety and “IR28’ has been reported to be salt sensitive. This comparison between the two genotypes supports the phenotypic data measurements taken at The Plant Accelerator®.

**Accession Name**	Accession no.	RGR.2to9 % decrease	EGRI.2to9
**Pokkali**	122206	18.9	0.81
**IR28**	122094	26.9	0.73

Supplementary Table 3: Phenotypic traits. List of phenotypic traits used in this study, with their abbreviation used in the text, their method of calculation and their units of measurement.

Serial number	Main trait	Trait/Index name	Abbreviation	Calculation	Unit	Adapted from
1	Relative Growth Rate (RGR)	Relative growth rate in control	RGRc	See METHODS section	day^{-1}	[1]
2		Relative growth rate in salt	RGRs		day^{-1}	[1]
3		Tolerance index	TOL	$RGR_c - RGR_s$	kpixels/day	TOL index[2]
4		Early growth response index	EGRI	RGR_s/RGR_c	-	Osmotic tolerance[3]
5		Stress-weighted performance	SWP	$RGR_s/\sqrt{RGRc}$	$\sqrt{kpixel/day}$	(Saade, Pillen, & Tester unpublished)
6		Stress susceptibility index	SSI	$(1-(RGR_s/RGR_c))/(RGRav_s/RGR_{avc})$	-	Stress susceptibility index[4]
7		Stress tolerance index	STI	$(RGR_c * RGR_s)/(RGR_{avc})^2$	-	Stress Tolerance Index[4]
8	Transpiration Rate (TR)	Transpiration rate in control	TR_c	See METHODS section	mL/day	
9		Transpiration rate in control	TR_s		mL/day	
10		Tolerance index in salt	TOL	$TR_c - TR_s$	mL/day	TOL index[2]
11		Salt/control	S/C	TR_s/TR_c	-	Salinity tolerance index
12		Stress-weighted performance	SWP	$TR_s/\sqrt{TRc}$	$\sqrt{mL/day}$	(Saade, Pillen, & Tester unpublished)
13		Stress susceptibility index	SSI	$(1-(TRs/TRc))/(TR_{avs}/TR_{avc})$	-	Stress susceptibility index[4]
14		Stress tolerance index	STI	$(TR_c * TR_s)/(TR_{avc})^2$	-	Stress Tolerance Index[4]
15	Transpiration Use Efficiency (TUE)	Transpiration use efficiency in control	TUE_c	See METHODS section	kpixels/mL	
16		Transpiration use efficiency in salt	TUE_s		kpixels/mL	
17		Tolerance index	TOL	$TUE_c - TUE_s$	kpixels/mL	TOL index[2]
18		Salt/control	S/C	TUE_s/TUE_c	-	Salinity tolerance index
19		Stress-weighted performance	SWP	$TUE_s/\sqrt{TUEc}$	$\sqrt{kpixel/mL}$	(Saade, Pillen, & Tester unpublished)
20		Stress susceptibility index	SSI	$(1-(TUE_s/TUE_c))/TUE_{avs}/TUE_{avc}$	-	Stress susceptibility index[4]

21	Stress tolerance index	STI	$(TUE_c * TUE_s)/(TUE_{avc})^2$	-	Stress Tolerance Index[4]

Supplementary Table 4: *indica* and *aus* accession codes.
Information about the accessions grown, including accessions number and name for *indica* and *aus*

indica

Seq.No	Acc.No	Acc.Name
1	120855	15577::IRGC 76987-1
2	120856	17/79/02-005::IRGC 51080-1
3	120859	849::IRGC 5970-1
4	121701	91-385::IRGC 63466-C1-G1
5	120860	AGAMI M 1::IRGC 4158-1
6	120861	AI LAN KE 1110::IRGC 67034-1
7	117636	AI-CHIAO-HONG
8	120862	AN QING ZAO::IRGC 72577-1
9	120863	ANAYANSI::IRGC 77474-1
10	121963	ANGIFOTSY 685::GERVEX 4717-C1
11	121705	APO::C1-G1
12	121965	ARANG::IRGC 43322-C1
13	120867	ARC 10818::IRGC 21079-1
14	121708	ARC 15872::IRGC 43249-C1-G1
15	120878	B 6144 F-MR-6::IRGC 117313-1
16	120879	BADA DHAN::IRGC 26540-1
17	121689	BADKALAMKATI::IRGC 45011-1
18	120880	BAI HE::IRGC 76437-1
19	120882	BALGALA GURMATIA::IRGC 61074-1
20	120883	BAMAWPYAN::IRGC 72458-1
21	120885	BANDIOUROU::IRGC 15980-1
22	120892	BG 301::IRGC 117315-1
23	120893	BG 34-11::IRGC 15782-1
24	121716	BH 2::C1-G1
25	117659	BINULAWAN
26	120895	BIRAIN 360::IRGC 6550-1
27	121717	BODOMANO::GERVEX 8343-C1-G1
28	121990	BOTRA MAITSO::GERVEX 8389-C1
29	121721	BOTRIKELY::GERVEX 8404-C1-G1
30	117623	BR24
31	120904	BYAT KYAR::IRGC 33004-1
32	121722	C 21::IRGC 331-C1-G1
33	122000	CARREON::IRGC 32575-C1
34	117443	CERE AIR::IRGC 43369-1
35	117681	CHANG CH'SANG HSU TAO
36	117682	CHAU
37	121725	CHERIVIRUPPU::IRGC 19928-C1-G1
38	120909	CHI TOU HUANG 1::IRGC 51280-1
39	117684	CHIEM CHANH
40	120912	CHINA 98-45-1::IRGC 1598-1
41	117447	CHITRAJ (DA 23)::IRGC 6208-1

42	120913	CHORUA KARTIKSAIL::IRGC 77230-1
43	120914	CHUA DAU::IRGC 4785-1
44	122011	CICA 8::C1
45	120916	CIMARRON::IRGC 116967-1
46	117691	CO 18
47	117454	CO 39::IRGC 51231-1
48	122025	CT 6510-24-1-2::C1
49	121237	DA 29 (SR 26 B)::IRGC 25850-1
50	120924	DA 5::IRGC 5855-1
51	117460	DA 9::IRGC 5854-1
52	120925	DA NUO (ZHAN)::IRGC 72024-1
53	121169	DALIFODE::IRGC 57766-1
54	122029	DANAU LAUT TAWAR::C1
55	117463	DE ABRIL::IRGC 50463-1
56	120929	DENG DENG QI::IRGC 72671-1
57	120939	DJOGOLON DJOGOLON::IRGC 75577-1
58	120946	E 5168::IRGC 68021-1
59	120947	E ZI 124::IRGC 70215-1
60	120952	EA HOUM::IRGC 12925-1
61	122042	ELONI::C1
62	120948	ELWEE::IRGC 15565-1
63	120949	EMBRAPA 6 CHUI::IRGC 116981-1
64	117470	FANDRAPOTSY::IRGC 10984-1
65	121663	GAJPATI::IRGC 58981-1
66	121738	GAMBIAKA KOKOUM::C1-G1
67	117478	GIE 57::IRGC 8231-1
68	120958	GODA HEENATI::IRGC 31393-1
69	120959	GOPAL::IRGC 61953-1
70	117745	GUAN-YIN-TSAN
71	122076	H 15-23-DA::C1
72	120964	HAO HOM::IRGC 12931-1
73	121743	HASAN SERAI::IRGC 79564-C1-G1
74	120970	HONG MI DONG MAO ZHAN::IRGC 68078-1
75	120972	HONG ZUI ZAO::IRGC 68090-1
76	122080	IAC 165::GERVEX 8508-C1
77	120977	ICTA POLOCHIC::IRGC 116997-1
78	120979	INIAP 415::IRGC 117001-1
79	122088	IR 1561-228-3-3::IRGC 32627-C1
80	122089	IR 19746-28-2-2::IRGC 78072-C1
81	122090	IR 20::C1
82	121748	IR 2006-P12-12-2::IRGC 32675-C1-G1
83	122091	IR 22::IRGC 11356-C1
84	122093	IR 2344-P1 PB-9-3-2B::IRGC 39317-C1
85	122094	IR 28::IRGC 30411-C1
86	120981	IR 31917-45-3-2::IRGC 78132-1
87	120982	IR 32453-20-3-2-2::IRGC 76331-1
88	117757	IR 36
89	120983	IR 43::IRGC 117005-1
90	121591	IR 5::IRGC 10321-1

91	121750	IR 50::C1-G1
92	121751	IR 52::IRGC 53434-C1-G1
93	122096	IR 53236-275-1::C1
94	121752	IR 55419-04::C1-G1
95	122098	IR 57920-AC 25-2-B::C1
96	122099	IR 57924-24::IRTP 16675-C1
97	120984	IR 59469-2B-3-2::IRGC 99703-1
98	121753	IR 60::IRGC 63493-C1-G1
99	121760	IR 72::C1-G1
100	120986	IR 74371-3-1-1::IRGC 117373-1
101	122112	IR 74371-54-1-1::C1
102	120987	IR 77298-14-1-2::IRGC 117374-1
103	120988	IR 77384-12-35-3-12-1-B::IRGC 117299-1
104	117758	IR 8
105	117268	IR64-21
106	120994	J 104::IRGC 117008-1
107	121771	JAMAJIGI::C1-G1
108	120998	JARIYU::IRGC 53265-1
109	121881	JC 120::IRGC 9178-1
110	117500	JC 91::IRGC 9177-1
111	117501	JC 92::IRGC 9176-1
112	121001	JINLING 78-102::IRGC 88421-1
113	121774	KALINGA III::C1-G1
114	121010	KANNI MURUNGA::IRGC 15432-1
115	121011	KARAYAL::IRGC 51001-1
116	121015	KATI::IRGC 67838-1
117	121016	KAUKHMWE::IRGC 33174-1
118	121019	KHAO DAW TAI::IRGC 24108-1
119	121235	KHAO DAWK MALI 105::IRGC 27748-1
120	121020	KHAO PON::IRGC 48114-1
121	117784	KIANG-CHOU-CHIU
122	121023	KINANDANG PUTI::IRGC 44513-1
123	121024	KIRIMURUNGA::IRGC 15585-1
124	122140	KOGONI 91-1::C1
125	121028	KUMBI::IRGC 752-1
126	117793	KUN-MIN-TSIEH-HUNAN
127	117520	LAGEADO::IRGC 50490-1
128	117521	LAL AMAN::IRGC 46202-1
129	121040	LIU XU::IRGC 74099-1
130	121785	LOHAMBITRO 224::GERVEX 5144-C1-G1
131	121041	LOKU SAMBA::IRGC 31462-1
132	121042	LONG ZI 1::IRGC 63726-1
133	121043	LUA TAU DUC::IRGC 16872-1
134	121044	MA GU ZI HE::IRGC 68212-1
135	121045	MA WAINE OHN::IRGC 33357-1
136	122156	MACAN BINUNDOK::IRGC 8245-C1
137	117525	MADAEL::IRGC 7722-1
138	121172	MAHADETHE::IRGC 74762-1
139	121420	MAHSURI::IRGC 10929-1

140	117527	MAKALIOKA 34::IRGC 6087-1
141	121049	MAKALIOKA::IRGC 77864-1
142	122159	MAMORIAKA 114::GERVEX 5176-C1
143	121050	MANDRIRAVINA::IRGC 69960-1
144	121794	MANGAVAVA FOTSILANSTSIKA 1177::GERVEX 5719-C1-G1
145	121051	MAURITIUS LOCAL::IRGC 5834-1
146	121052	MENAKELY::IRGC 69963-1
147	121053	MG 2::IRGC 79837-1
148	117531	MILYANG 23::IRGC 34393-1
149	117271	Minghui 63
150	121059	MOTTA SAMBA::IRGC 36489-1
151	117533	MTU 9::IRGC 7919-1
152	117817	MTU9
153	121062	NAKABAWA::IRGC 70676-1
154	122178	NAM ROO::C1
155	122179	NAM SA GUI 19::IRTP 6892-C1
156	121063	NAN TE 113::IRGC 70345-1
157	121064	NARGUNI::IRGC 74713-1
158	121065	NARU HATHIYAL::IRGC 15547-1
159	121066	NCS 130::IRGC 51879-1
160	121576	NGAJA::IRGC 64917-1
161	121799	NIONOKA::C1-G1
162	122181	NONA BOKRA::IRGC 22710-C1
163	121070	NORUNKAN::IRGC 8934-1
164	121073	NS 113::IRGC 68838-1
165	121074	NS 1611::IRGC 68963-1
166	117826	O-LUEN-CHEUNG
167	117829	ORYZICA LLANOS 5
168	121632	ORYZICA SABANA 10::IRGC 117018-1
169	121077	PANAKALI::IRGC 47399-1
170	117840	PAO TOU HUNG
171	117841	PAPPAKU
172	121079	PATCHAIPERUMAL::IRGC 15681-1
173	117555	PATIK::IRGC 43530-1
174	121080	PAWHTUN::IRGC 33562-1
175	121804	PCT 11\0\0\2,BO 1>55-1-3-1::C1-G1
176	121807	PEH PI NUO::IRGC 8266-C1-G1
177	117848	PEH-KUH
178	117849	PEH-KUH-TSAO-TU
179	121889	PELITA JANGGUT::IRGC 43540-1
180	121083	PERUM KARUPPAN::IRGC 15524-1
181	117559	PETA::IRGC 32571-1
182	121085	PICONEGRO::IRGC 117022-1
183	117560	PIN KAEO::IRGC 5803-1
184	117561	PIN TAWNG::IRGC 40673-1
185	121087	PINURSIGI::IRGC 26889-1
186	122206	POKKALI::IRGC 108921-C1
187	121808	POPOT::IRGC 43545-C1-G1
188	121089	PSBRC 18::IRGC 117375-1

189	117563	PTB 25::IRGC 6386-1
190	117564	PTB 9::IRGC 6274-1
191	121091	PURBIA (KALANSAR)::IRGC 59189-1
192	121092	QING GU::IRGC 59839-1
193	121093	QING SHUI ZAO::IRGC 72807-1
194	121094	RACE::IRGC 15706-1
195	121095	RAJ BHOG::IRGC 77294-1
196	121098	RATHKANDIRAM::IRGC 36507-1
197	117567	RATHUWEE::IRGC 8952-1
198	121099	RAY JAZAYKAYZ::IRGC 62181-1
199	121100	RAY NABJA::IRGC 62184-1
200	121101	RED PIE BOLD 17-214::IRGC 38207-1
201	121811	ROJOFOTSY 693::GERVEX 8407-C1-G1
202	121812	ROJOMENA 1034::GERVEX 8412-C1-G1
203	121102	RR 166-645::IRGC 117352-1
204	117622	RTS 14
205	117574	RTS 4::IRGC 8177-1
206	121104	RTS 5::IRGC 8233-1
207	121105	RUZZ (HABUR)::IRGC 55679-1
208	121106	SADA DANGA BORO::IRGC 77298-1
209	117276	Sadu Cho
210	122232	SAHEL 108::C1
211	121942	SAHEL 159::C1-G1
212	121821	SAHELIKA::C1-G1
213	122236	SALUMPIKIT::IRTP 4777-C1
214	122239	SAMBALA MALO::C1
215	121110	SAN DU BAI MI HONG GU::IRGC 59849-1
216	121111	SAN RI QI::IRGC 59855-1
217	121639	SAO::IRGC 61467-1
218	121945	SATHI 34-36::C1-G1
219	117880	SERATOES HARI
220	117881	SHAI-KUH
221	117277	Shan-Huang-Zhan-2
222	121118	SHONTH::IRGC 74717-1
223	121122	SOKOU MALSIRA::IRGC 77301-1
224	121642	SOM CAU 70 A::IRGC 8227-1
225	121124	SOMIMADAMO::IRGC 69044-1
226	121823	SOMIZY::GERVEX 8419-C1-G1
227	122255	SONA::IRGC 26971-C1
228	121127	SUDUWEE::IRGC 8972-1
229	121130	SURMANIYA::IRGC 61148-1
230	121131	SUTHUWEE::IRGC 8915-1
231	122258	SWARNA::IRGC 117278-C1
232	121133	T 26::IRGC 46768-1
233	117907	TAICHUNG NATIVE 1
234	117587	TD 25::IRGC 9146-1
235	121827	TELIMANI::C1-G1
236	121136	TELOVOLANA::IRGC 69969-1
237	117912	TEQING

238	117589	TETEP::IRGC 32576-1
239	121225	THAPACHINIYA::IRGC 16234-1
240	121137	TI KU::IRGC 1224-1
241	117915	TKM 6
242	121139	TNAU 7456::IRGC 39858-1
243	121829	TOKAMBANY 663::GERVEX 8358-C1-G1
244	121830	TOKAMBANY 669::GERVEX 8406-C1-G1
245	121140	TSIAMPOETRY::IRGC 77902-1
246	121834	TSIPALA FOTSY 1883::GERVEX 5387-C1-G1
247	121141	TSIPALA FOTSY::IRGC 69973-1
248	121142	TSIPALA MENA::IRGC 69977-1
249	121144	TSIPALA::IRGC 10989-1
250	121145	TUNG CH'IU AI::IRGC 34265-1
251	121146	UBA MURALI::IRGC 25928-1
252	121147	UP 1537::IRGC 70490-1
253	121835	UPL RI 5::IRTP 7034-C1-G1
254	122272	UPL RI 7::IRTP 9897-C1
255	117598	VANDANA::IRGC 117398-1
256	121836	VARY MADINIKA 3494::GERVEX 8318-C1-G1
257	121839	VARY VATO 154::GERVEX 5429-C1-G1
258	121151	VARY VATO MENAHODITRA::IRGC 69111-1
259	121840	VATO MATSOAMALONA::GERVEX 8454-C1-G1
260	121152	VELLAI SEENETTI::IRGC 15516-1
261	121844	WAB 706-3-4-K4-KB-1::C1-G1
262	121153	WANNI DAHANALA::IRGC 15721-1
263	121846	WAS 169-B-B-4-2-1::C1-G1
264	122284	WAS 170-B-B-1-1::C1
265	122285	WAS 173-B-B-6-2-2::C1
266	122286	WAS 174-B-3-5::C1
267	121847	WAS 181-B-6-3::C1-G1
268	121848	WAS 182-B-1-1::C1-G1
269	121849	WAS 183-B-6-2-3::C1-G1
270	121850	WAS 194-B-3-2-5::C1-G1
271	121851	WAS 197-B-6-3-11::C1-G1
272	122287	WAS 198-B-3-1-3::C1
273	122288	WAS 199-B-1-2-1::C1
274	121852	WAS 200-B-B-1-1-1::C1-G1
275	121853	WAS 202-B-B-1-1-2::C1-G1
276	122289	WAS 203-B-B-2-4-1::C1
277	122290	WAS 206-B-B-2-2-1::C1
278	121854	WAS 207-B-B-3-1-1::C1-G1
279	121855	WAS 208-B-B-5-1-1-3::C1-G1
280	121856	WAS 20-B-B-1-2-2::C1-G1
281	122291	WAS 21-B-B-20-4-3-3::C1
282	121857	WAS 30-11-4-6-2-2-1::C1-G1
283	122292	WAS 33-B-B-15-1-4-5::C1
284	121858	WAS 62-B-B-17-1-1-3::C1-G1
285	122297	WAS 63-22-5-9-10-1::C1
286	122298	WAY RAREM::IRTP 23013-C1

287	121156	XI GU HONG::IRGC 74226-1
288	121176	XI GU ZAO::IRGC 72360-1
289	121157	XI NUO ZAO::IRGC 68279-1
290	121158	YAKADA::IRGC 51096-1
291	121159	YE TI ZHAN::IRGC 68296-1
292	121161	ZALCHA::IRGC 62190-1
293	121162	ZAO SHAO ZHAN::IRGC 68318-1
294	121163	ZAO SHOU 691-11::IRGC 70447-1
295	117280	Zhenshan 97B
296	121164	ZI GAN NAN GU::IRGC 70468-1
297	121165	ZS 4::IRGC 56707-1

aus

Seq.No.	Acc.No	Acc.Name
1	28425	182
2	28364	29 A-2
3	7738	ANDIKULAN
4	56562	ANGALAIS
5	74720	ANOOPA
6	20709	ARC 10100
7	12386	ARC 10177
8	12430	ARC 10317
9	42572	ARC 10319
10	12440	ARC 10352
11	20887	ARC 10376
12	12683	ARC 10955
13	21477	ARC 11559
14	21618	ARC 11755
15	21634	ARC 11772
16	21780	ARC 11959
17	41001	ARC 12071
18	41391	ARC 14088
19	42019	ARC 15340
20	12282	ARC 6578
21	20499	ARC 7099
22	12331	Arc 7229
23	33983	ASAHI
24	6591	Aswina 330
25	29016	*aus* 196
26	29049	*aus* 257
27	28904	*aus* 41
28	29221	*aus* 439
29	28918	*aus* 55
30	28934	*aus* 71
31	25833	*aus* JHARI
32	66767	*aus* JOTA
33	61909	BAGETAULI
34	25835	BAILAM
35	12884	Bala (CR 42-38)
36	6538	BAMOIA 341

37	74728	BANS 4
38	27509	BARAN BORO
39	27798	Basmati 1
40	25838	BATHURI
41	64766	BAULAN
42	34737	BAWOI
43	33990	BEGUM
44	25839	BENAFUL
45	25840	BENAMURI
46	6588	BHADOIA 303
47	6551	BHADOIA 685
48	66830	BHORIA *aus*
49	74733	BIRANJ
50	3711	BJ1
51	40275	Black Gora (NCS 12)
52	49022	BOILAN
53	31572	BOLIUM
54	34682	BOTESHAWAR
55	78238	BRE JER
56	45234	BROWN GORA
57	6349	CA 902-B-2-1
58	25845	CHANDARHAT
59	73091	CHAWAL
60	77228	CHENGRI 2
61	66774	CHENGRI MURALI
62	52101	CHIADI NAKI
63	64771	CHIKON SHONI
64	17052	Chuan 4
65	25855	CHUNGUR BALI
66	45374	CN10183-S/C-244
67	45336	CROSS 4-244
68	8704	CTG 1516
69	6246	DA28
70	6422	DA8
71	58943	DAINY
72	25849	DAL KASHAI
73	76296	DANGAR
74	8626	DD 62
75	74737	DEHULA
76	66776	DHAL KACHAI
77	77236	DHALA BAGDAR 2
78	66779	DHALA BHADOI
79	25851	DHALASHAITA
80	60530	DHALI KHAMA
81	3681	DHAN
82	64773	DHARIA
83	64774	DHARIA BOALIA
84	64775	DHEKI SHAITA
85	27513	DHOLI BORO

86	8455	DJ 123
87	8506	DJ 24
88	8581	DK 12
89	8769	DM 43
90	8787	DM 56
91	8428	DNJ 140
92	16261	DUDHI SAROO
93	636	DULAR
94	32561	DULAR
95	25852	DUMAI
96	8861	DV 123
97	8839	DV85
98	8517	DZ193
99	8555	DZ78
100	36148	ETYM
101	117267	FR 13 A
102	6144	FR 13A
103	8887	FR13 A
104	66783	FULKATI
105	73098	GADRA
106	73099	GADRA
107	25854	GARIA
108	32300	GERDEH
109	58278	GHATI KAMMA NANGARHAR
110	19740	GO CHEON DO
111	49189	GOAI
112	66787	Gochi Boro
113	66791	GORBAI
114	66792	GUL MURALI
115	8961	H4
116	10873	Habigonj Boro 6
117	74744	HARBHOONDI
118	14737	Harlan No. 25A
119	16817	HASAWI
120	8942	HEENBALAWEE
121	52441	HEGRA
122	67631	HODARAWALA
123	64778	HOLOI BASH(SOLOI BASH)
124	53989	IC27525
125	77250	IKRA
126	25865	JABARSHAIL
127	66831	JABOR SAIL
128	66861	JABOR SAIL
129	27516	Jagli Boro
130	73101	JAMBALI
131	73102	JAMBALI BUSSA
132	37117	JAMRI
133	9069	JC148
134	27967	JHONA 26

135	3742	JHONA 349
136	6307	JHONA 349
137	25867	JHUM BEGUNBICHI
138	25868	JHUM FULBADAM
139	25874	JHUM SONALICHIKON
140	66800	JUMA
141	64780	KAL SHONI
142	34959	KALA SANI
143	45975	KALAMKATI
144	77201	KALIBORO
145	66806	KALO KUCHI
146	49746	KALU A. 30
147	7702	KALUBALA VEE
148	7755	Kalukantha
149	67019	KAMOZ
150	73105	KANGRO
151	6618	Karkati 87
152	47381	KARUTHA CHEENEDDI
153	74751	KASAPUR
154	64781	KAT GIMI
155	9007	Kaukau
156	34983	KELE BARDHAN
157	66807	KELEE
158	54072	Khadasiya 3 (Straw)
159	27748	KHAO DAWK MALI 105
160	66808	KHARAI MURALI
161	28016	KHARSU 80
162	28598	KOALARETA
163	66811	KOI MURALI
164	77267	KOYRA
165	15449	KURKARUPPAN
166	66518	KURULU WEE(WHITE)
167	34712	LAKHI PURI
168	25885	LAKHSNIKAJAL
169	43915	LALSAITA
170	66815	LENJA MURALI
171	27762	Leuang Pratew
172	29374	MADHABSAIL 741
173	51021	MAHA PANNITHI
174	6427	MAHLAR 346
175	52696	MAHSURI
176	12883	Mehr
177	61011	MEKRA BHOL
178	34722	MERY
179	25888	MICOCHU
180	25893	MIKOTCHU
181	25901	MIRITI
182	27571	MI-TIMBRA
183	25906	MOLLADIGHA

184	66817	MOSHIA BHADOI
185	64789	MOSHUR
186	66818	MOYNA MOTI
187	66819	MUNSHI MURALI
188	36333	MUTTU SAMBA
189	4819	N 22
190	6264	N 22
191	19379	N 22
192	46458	N 22
193	46459	N 22
194	117273	N 22
195	64792	NARIKEL JHUPI
196	51903	NCS160
197	51923	NCS183
198	62530	NCS840
199	31611	NOROI
200	32559	NP125
201	68925	NS 1254
202	28134	P 660
203	28171	P 737
204	27209	PAEDAI MESARAHA
205	25911	PANKHIRAJ
206	66527	PANNITI
207	66526	PANNITTI
208	6252	Paung Malaung C 3
209	32571	PETA
210	32399	PHUDUGEY
211	36345	PODI HEENATI
212	6304	PTB30
213	77530	Puluthi Karan (Mixed)
214	14487	RADIN PAHANG
215	64793	RAKHOIL
216	66822	RANGPURI *aus*
217	77210	RAYADA
218	77298	Sada Danga Boro
219	73118	SADA SOLAY
220	74775	SAFED MACAN
221	25920	SAMPATTI
222	28212	SANTHI SUFAID 207
223	67757	SAREINA
224	28229	SATHI
225	28239	SATHRA 278
226	74716	SAYARI
227	47414	SEENETTI
228	34732	SERETY
229	64796	SHADA SHAITA
230	74777	SHANKAR
231	14528	SHIRKATI
232	35154	SIMUL KHURI

233	5418	SINTANE DIOFOR
234	63	SLO 16
235	52763	SOKANA
236	73121	SOLAY GHAT
237	37598	SOLOI
238	64799	SREERAMPUR SHAITA
239	28303	SUFAID 246
240	25924	SULTANJATA
241	35166	SURJA MUKHI
242	8256	SURJAMKUHI
243	6294	T 1
244	46768	T 26
245	73124	TAK
246	73126	TAK SIAH
247	73127	TAK SUFAID
248	13746	TAOTHABI
249	32362	TCHAMPA
250	25925	TEPAKAIN
251	27519	TEPI BORO
252	15325	THAVALU
253	44229	UPRB56
254	61631	UPRH184
255	74719	URYEE BOOTA
256	67661	WEDA HEENATI

Supplementary Table 5: Genotypic and phenotypic information for the two different GWAS models used.

Two sets of genotypic information were used for the *indica* panel - 'GBS 40K SNP' and '700K SNP' - with their respective number of accessions and total number of SNPs. One set of genotypic information was used for the *aus* panel. The total number of accessions phenotyped is also given. Model 1 refers to the conventional MLM model and model 2 refers to the Interaction model.

	indica		*aus*		*INDAUS*	
**Genotypic data**	Accessions	Total SNPs	Accessions	Total SNPs	Accessions	Total SNPs
**GBS 40K SNP**	274	17,924	-	-	-	-
**700K SNP (model 1)**	228	397,659	146	394,786	374	304,877
**700K SNP (model 2)**	228	19,436	146	16,538	372	5,560

Supplementary Table 6: Summary of candidate genes and local linkage disequilibrium (LD) region underlying SNPs from the suggestive threshold $P = 10^{-5}$ [5,6] for transpiration use efficiency (TUE) using the mixed linear model (MLM). All the significant associations are presented for the three diversity panels (*indica*, *aus* and *INDAUS)* and intervals (2-9; 2-6; 6-9; 9-13 days after treatment)

Model	Chrom.	Population	Trait	Time interval for days after treatment	QTL region (bp)	SNP ID	*P*-value	Candidate gene
**Conventional MLM**	3	*Indica*	TUE (TOL)	2-9	32593832..32595034	SNP-3.32587904.	8.72×10^{-6}	Os03g57160 (Zinc ion binding protein)
			TUE (TOL)	2-6	32595034..32602359	in LD region		Os03g57170 (cell cycle control protein)
			TUE (S/C)	6-9	27042766..27050145	in LD region		Os03g47720 (Ankyrin)
			TUE (TOL)	6-9	13135925	SNP-3.13134642.	6.70×10^{-6}	Os03g22740 (nucleolar protein NOP5-1)
		INDAUS	TUE (S/C)	6-9	26991428..27012332	in LD region		Os03g47650 (Ankyrin)
						in LD region		Os03g47670(Ankyrin)
						SNP-3.27005384.	4.20×10^{-6}	Os03g47686(Ankyrin)
					27046828..27054059	SNP-3.27039880.	7.25×10^{-6}	Os03g47720 (Ankyrin)
						in LD region		Os03g47730 (homeobox domain containing protein)
	11	*Indica*	TUE (TOL)	2-6	3638272..3641726	in LD region		Os11g07230 (receptor kinase)
						SNP-11.3637627.	9.42×10^{-6}	Os11g07240 (serine/threonine-protein kinase BRI1-like 2 precursor)
			TUE (TOL)	2-6	3509758	SNP-11.3505660.	5.30×10^{-7}	Os11g07050 (FHA domain containing protein)

			TUE (TOL)	2-9				
			TUE (TOL)	6-9				

1 Hunt, R., Causton, D. R., Shipley, B. & Askew, A. P. A modern tool for classical plant growth analysis. *Ann Bot-London* **90**, 485-488 (2002).

2 Rosielle, A. A. & Hamblin, J. Theoretical aspects of selection for yield in stress and non-stress environments. *Crop Sci* **21**, 943-946 (1981).

3 Berger, B., de Regt, B. & Tester, M. Trait dissection of salinity tolerance with plant phenomics. *Methods Mol. Biol.* **913**, 399-413, doi:10.1007/978-1-61779-986-0_27 (2012).

4 Fernandez, G. C. J. in *International Symposium on Adaptation of Food Crops to Temperature and Water Stress.* (ed CG Kuo) 257-270 (Asian Vegetable Research & Development CTR).

5 Crowell, S. *et al.* Genome-wide association and high-resolution phenotyping link Oryza sativa panicle traits to numerous trait-specific QTL clusters. *Nat Commun* **7**, 10527, doi:10.1038/ncomms10527 (2016).

6 Rebolledo, M. C. *et al.* Phenotypic and genetic dissection of component traits for early vigour in rice using plant growth modelling, sugar content analyses and association mapping. *Journal of Experimental Botany* **66**, 5555-5566 (2015).
