# Supplementary figures and images for: Salinity tolerance loci revealed in rice using high-throughput non-invasive phenotyping
Source: Nat Commun. 2016 Nov 17;7:13342. doi: 10.1038/ncomms13342 (PMC5118543; doi:10.1038/ncomms13342)

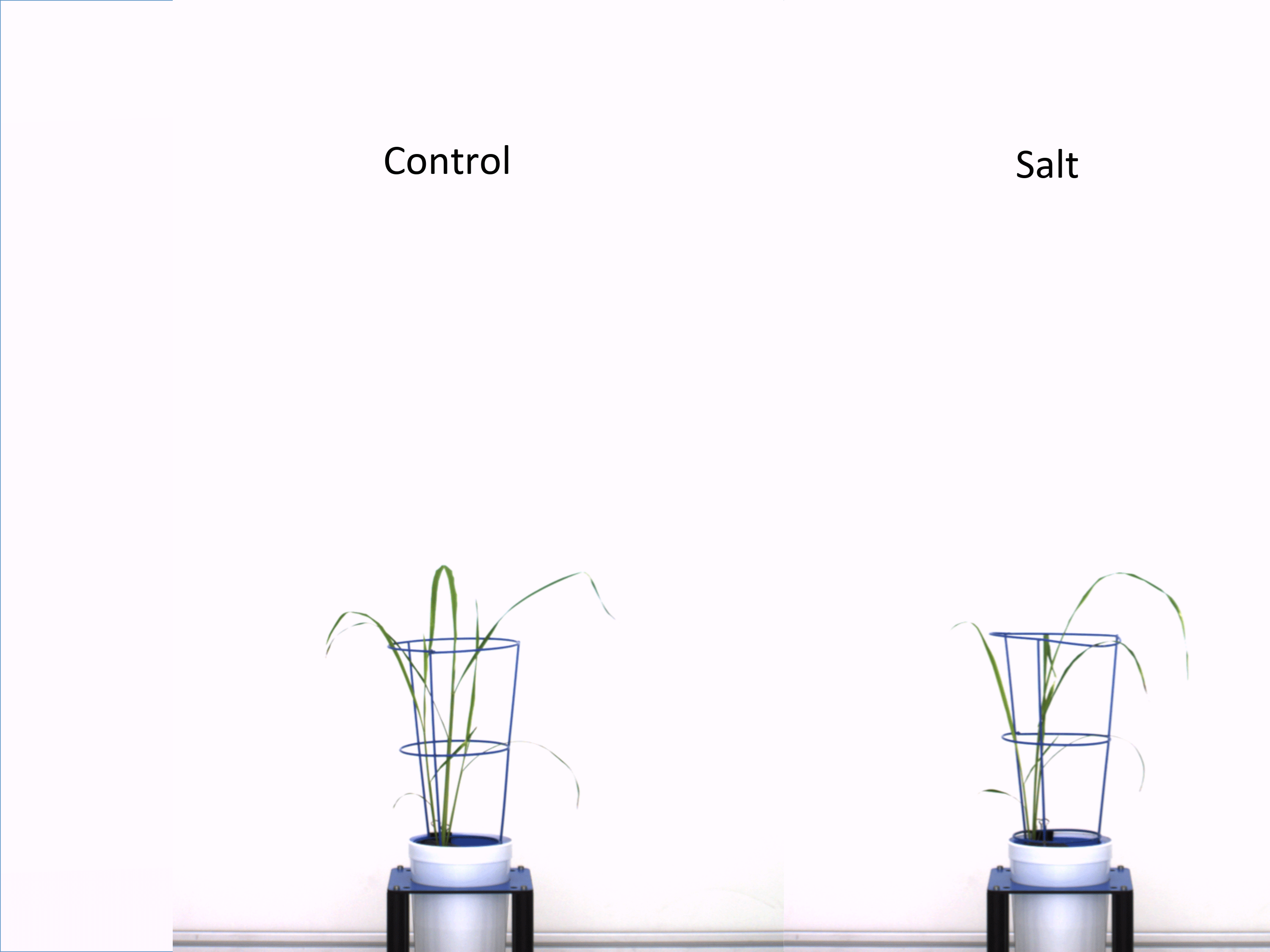

Supplement: Supplementary Movie 1 — Time lapse movie of two rice plants of the same accession growing over 13 days, one in control conditions and one under saline conditions. [file ncomms13342-s2.gif]
